# Cervical cancer burden and elimination readiness in the US-affiliated Pacific Islands: A structured narrative review

**DOI:** 10.1016/j.canep.2026.103144

**Published:** 2026-06-29

**Authors:** Diep Thi Ngoc Nguyen, Rubana Islam, Jin Qin, Virginia Senkomago, Lee Buenconsejo-Lum, Youngju Jeong, Sameer V. Gopalani, Yoon-Jung Kang, Michael David, Martina Reichhardt, James Edilyong, Ashley Tippins, Enriquito Lu, Emi Chutaro, Isabel Scarinci, Theresa Arriola, Kate Simms, Karen Canfell, Neal Palafox

**Affiliations:** a Cancer Elimination Collaboration, School of Public Health, Faculty of Medicine and Health, the University of Sydney, Sydney, Australia; b Australian Institute of Health Innovation, Centre for Health Informatics, Macquarie university, Australia; c Division of Cancer Prevention and Control, Centers for Disease Control and Prevention, Atlanta, United States; d John A. Burns School of Medicine, University of Hawaii, United States; e Department of Health Care Management, College of Health Sciences, University of Sharjah, United Arab Emirates; f Daffodil Centre, Sydney, Australia; g School of Medicine & Dentistry, Griffith University, Gold Coast, Australia; h Yap State Department of Health Services, Federated States of Micronesia, Federated States of Micronesia; i Guam Department of Public Health and Social Services, Guam; j Immunization Services Division, Centers for Disease Control and Prevention, Atlanta, United States; k Pacific Island Health Officers' Association (PIHOA), Hawai'i; l O’Neal Comprehensive Cancer Center, University of Alabama at Birmingham, United States; m Independent Consultant of PACe (Pacific Against Cervical Cancer) project, United States

## Abstract

**Background:**

The US-Affiliated Pacific Islands (USAPI) experience a high burden of cervical cancer, but progress toward achieving cervical cancer elimination targets is difficult to assess due to limited and fragmented data on HPV vaccination, cervical cancer screening and cancer treatment access. This narrative review aims to synthesise evidence of disease burden, risk factors, and interventions to inform region-specific strategies.

**Methods:**

We conducted a structured narrative review of published literature (MEDLINE, Embase, Global Health), cancer registry data, surveillance reports, and health system registries for six USAPI jurisdictions from 1990 to 2025. We collated information on HPV, cervical precancers and cancers, and related risk factors. Information on HPV vaccination, cervical cancer screening, and treatment, obtained from jurisdictional health system registries and reports, was used to assess the region’s trajectory towards the elimination targets.

**Findings:**

Cervical cancer incidence among women aged ≥ 20 years during 2007–2022 in the USAPI (age-standardized rate:17.2/100,000;95%CI:15.6–18.8) exceeded that of the US (10.8/100,000;95%CI:10.7–10.8), with large variation across jurisdictions, from 8.4/100,000 in American Samoa to 68.9/100,000 in Republic of the Marshall Islands (RMI). Approximately 75% of cases were diagnosed at Stage III + . HPV vaccination was introduced between 2007–2016, with coverages ranging from 44.2% in Federated States of Micronesia (FSM) to 92.7% in Commonwealth of the Northern Mariana Islands (CNMI). Four jurisdictions have implemented cytology-based screening, whereas RMI/FSM primarily rely on visual inspection with acetic acid. Screening coverage varies from 14.2% (CNMI, 2019) to 67.8% (Guam, 2020). Cancer treatment capacity is limited, with most jurisdictions lacking radiotherapy and relying on off-island referrals for advanced cases.

**Interpretation:**

USAPI cervical cancer burden is substantially higher than the US, compounded by late-stage diagnoses and limited treatment capacity. Although vaccination coverage has improved, major gaps remain in screening, follow-up, and access to care. Strengthening health systems through scalable, resource-appropriate strategies will accelerate progress toward elimination.

## 1. Introduction

Cervical cancer is the fourth most common cancer among women worldwide. The age-standardized rates (ASR) of cervical cancer incidence and mortality were estimated at 13.3 and 7.3 per 100,000 women, respectively, equivalent to an estimated 604,127 new cases and 341,831 deaths in 2022 [1]. Approximately 88% of cases and 91% of cervical cancer-related deaths occur in low- and middle-income countries. (1) Persistent infection with oncogenic human papillomavirus (HPV) types is responsible for approximately 90% cervical cancer cases [2]. Additionally, the prevalence of high-grade squamous intraepithelial lesions (HSIL), which is strongly linked to persistent infection with high-risk HPV types, is considered a precursor that can precede to invasive cervical cancer if left untreated [3]. Furthermore, it is important to understand risk factors that are likely to be associated with the transmission of HPV (correlates of HPV infection) and the progression of HPV infection to cervical cancer (cofactors for HPV progression). According to findings from international pooled analyses, certain types of sexual behaviour, including early sexual debut, multiple lifetime sexual partners, and exposure to sexually transmitted infections (STIs) have been identified as correlates of HPV infection. Factors associated with progression of HPV infection to cervical cancer, such as oral contraceptive use, smoking, parity, and age at first full-term pregnancy, are referred to as cofactors for cervical cancer progression [4–6].

In 2020, the World Health Organization (WHO) launched a global strategy to eliminate cervical cancer by establishing 2030 targets for vaccinating 90% of girls by the age of 15 years, screening 70% of eligible women at least twice in their lifetimes using a high-performance test (currently only human papillomavirus [HPV] testing), and offering adequate treatment and care for 90% of women with pre-cancer and invasive cancer (90–70–90 targets) [7]. In 2022, the first-dose HPV vaccine program coverage rates in high-income countries were estimated at 56% in girls and 42% in boys, respectively [8]. In contrast, this coverage is estimated to be 16% in girls by age 15 years in lower-middle income countries [8]. In 2019, in low- and middle-income countries where the burden of cervical cancer remains highest, it was estimated that less than 20% of women received cervical cancer screening in their lifetime, compared to 48% estimated in high-income countries [9]. Limited access to cancer treatment services, such as radiotherapy, has also been reported in low- and middle-income countries [10].

The US-Affiliated Pacific Islands (USAPI), encompassing three freely associated states (i.e., Federated States of Micronesia [FSM], Republic of the Marshall Islands [RMI], and the Republic of Palau) and three US territories, including Guam, American Samoa, and the Commonwealth of the Northern Mariana Islands (CNMI), constitute a unique political and geographic region in the Pacific Ocean. Among the six jurisdictions, only Guam and CNMI are categorised as high-income economies, whereas the remaining four are characterised as lower- or upper-middle-income economies, with GDP per capita ranging from US$3464 in FSM to US$35,600 in Guam [11]. The small population of approximately 470,000 in USAPI is dispersed across 5 million square miles of the Pacific Ocean, making the effective geographic reach of essential services difficult [12]. Besides economic disadvantage, these island nations struggle with access to quality health care, with only the main islands having access to more comprehensive health infrastructure and trained medical staff [12]. Generally, USAPI jurisdictions are medically underserved because of a shortage of health care providers, and as a result, the effective delivery of services for vaccination, screening, diagnosis, and treatment of cancer is challenging [13].

Cervical cancer incidence and mortality data for jurisdictions in the USAPI have not been reported by the international databases, including the Global Cancer Observatory (GLOBOCAN) and the International Agency for Research on Cancer (IARC), except Guam. Most recently, a study was conducted to report the cervical cancer incidence and mortality based on 2007–2020 cancer registry data in the USAPI [14]. The age-standardised incidence rate of cervical cancer of 21.7–22.1 per 100, 000 women (age ≥20 years) was estimated for the USAPI with the variation among jurisdictions [14]. Two separate studies reported on HPV vaccination coverage [15] and cervical cancer screening coverage [16,17] in this region. However, more updated data from cancer registries for the USAPI is now available and a comprehensive review of cervical cancer, its risk factors, and current interventions has not been conducted to identify the existing gaps, to update the knowledge base, and to provide a contextual analysis to support future cervical cancer elimination initiatives in the USAPI [14]. Therefore, in this paper, we aim to narratively review the available data on the overall burden of cervical cancer, its correlates and cofactors, and to review the progress in the USAPI towards meeting the WHO 2030 90–70–90 targets for cervical cancer elimination.

## 2. Materials and methods

Relevant review methods have been applied to address specific themes aligning with specific research questions. Firstly, a literature review method was applied to review the prevalence of HPV infection and HSIL among the general female population and the associated risk factors in the USAPI. Secondly, regional and international cancer statistics databases have been purposely reviewed for cervical cancer incidence and mortality data. Thirdly, health information systems, surveillance reports, regional cancer registry reports have been purposely selected for review and extraction of data regarding cervical cancer prevention and control interventions in the USAPI. (Figure A2, Appendix) Two reviewers (RI and DN) independently conducted the search, screening and selection in the first stage. Any disagreements were resolved by discussing with the third reviewer (KS).

### 2.1. Prevalence of HPV infection, precancerous lesions, and correlates and cofactors related to the progression of cervical cancer in the USAPI

We reviewed published literature, survey reports, and grey literature reports of six jurisdictions to identify the data on the prevalence of HPV infections and high-grade precancerous lesions among the general female population, and correlates and cofactors associated with HPV infection and progression of cervical cancer.

We developed separate search strategies using key terms for HPV infection and HSIL, and correlates of HPV infection and cofactors of progression from HPV infection to cervical cancer in USAPI (Search Strategy, Appendix). MEDLINE, Embase, and Global Health databases from January 1990 to July 2025 were systematically screened. In addition, complementary search activities, including citation chaining and searching, were conducted to identify relevant articles.

Articles were included if they reported prevalence estimates of the burden of disease, including the prevalence of HPV infection (any HPV or high-risk HPV) and HSIL among the general female population, and cervical cancer risk factors in adolescents or adults. For prevalence of cervical HPV infection and HSIL, population-based cross-sectional studies were included. For correlates and cofactors associated with the transmission of cervical HPV infection and cervical cancer progression, cohort and nested case-control studies were the best study designs in the inclusion criteria; if none of these study designs were identified, population-based cross-sectional studies were included. We also included systematic reviews that reported the outcomes of interest and ensure including any additional primary studies that did not appear from the search to avoid double-counting. We excluded studies that did not report any of the outcomes of interest, were in a language other than English, and articles that were editorials or comments. (See Inclusion and exclusion criteria in Table A2 and A3, Appendix)

Aside from searching electronic databases and to maximize the inclusion of relevant data, we looked at grey literature sources. Human subjects review was not required because only data without identifiers were used. These sources included in-country resources, such as Ministry of Health and statistical bureau documents; materials published by the US-based Department of Health and Human Services, including by the Centers for Disease Control and Prevention (CDC); regional resources, such as the Pacific Island Health Officers Association (PIHOA); We also used survey data obtained from the Behavioral Risk Factor Surveillance System (BRFSS), National Vital Statistics System of the United States, Global Youth Tobacco Survey (GYTS), Youth Risk Behavior Surveillance System (YRBSS), Rapid Youth Survey (YRS), and Non-Communicable-Disease (NCD) Adult Hybrid Survey over multiple years.

Citations were managed in Endnote (EndNote v18, Vancouver, Philadelphia, Pennsylvania, United States), and duplicates were removed before title and abstract screening. Full text screening and data extraction were conducted in parallel by using a template in Excel (Excel, Version 2409, Microsoft Corporation, Redmond, Washington, United States). This included study characteristics, participant demographics, outcomes measured, and results. The data were synthesized according to themes aligning with the review questions. Because of a paucity of appropriate quantitative data for each jurisdiction and the USAPI overall, meta-analyses were not appropriate, and therefore, not conducted.

### 2.2. Cervical cancer incidence and mortality

We reviewed cervical cancer incidence and mortality information from the Pacific Regional Cervical Cancer Registration (PRCCR) and international databases, including *Cancer Incidence in Five Continents* (CI5), the International Agency Research for Cancer (IARC), and the Global Cancer Observatory (GLOBOCAN) database; and the available peer-reviewed literature has also been reviewed. Incidence data from the PRCCR were obtained from the most recent PRCCR report (2007–2022) [18]. Mortality data were obtained from the PRCCR deidentified dataset (2007–2022) [19].

### 2.3. Current status of cervical cancer prevention and control in the USAPI

For HPV vaccination coverage, we used data from the jurisdictional immunization information system (IIS), the National Immunization Surveillance Teen reports, and the WHO immunization portal. For cervical cancer screening coverage, we extracted data from the Health Center Program Uniform Data System (UDS), and BRFSS and the NCD Adult Hybrid Survey reports. For an assessment of treatment capacity for cervical precancer and cancer, information from Cancer in the US-Affiliated Pacific Islands, 2007–2022 report was extracted [18]. Additional information on precancer and cancer treatment have been gathered and verified through discussions with local partners and the experience of the PACe project.

## 3. Results

**Fig. 1.**
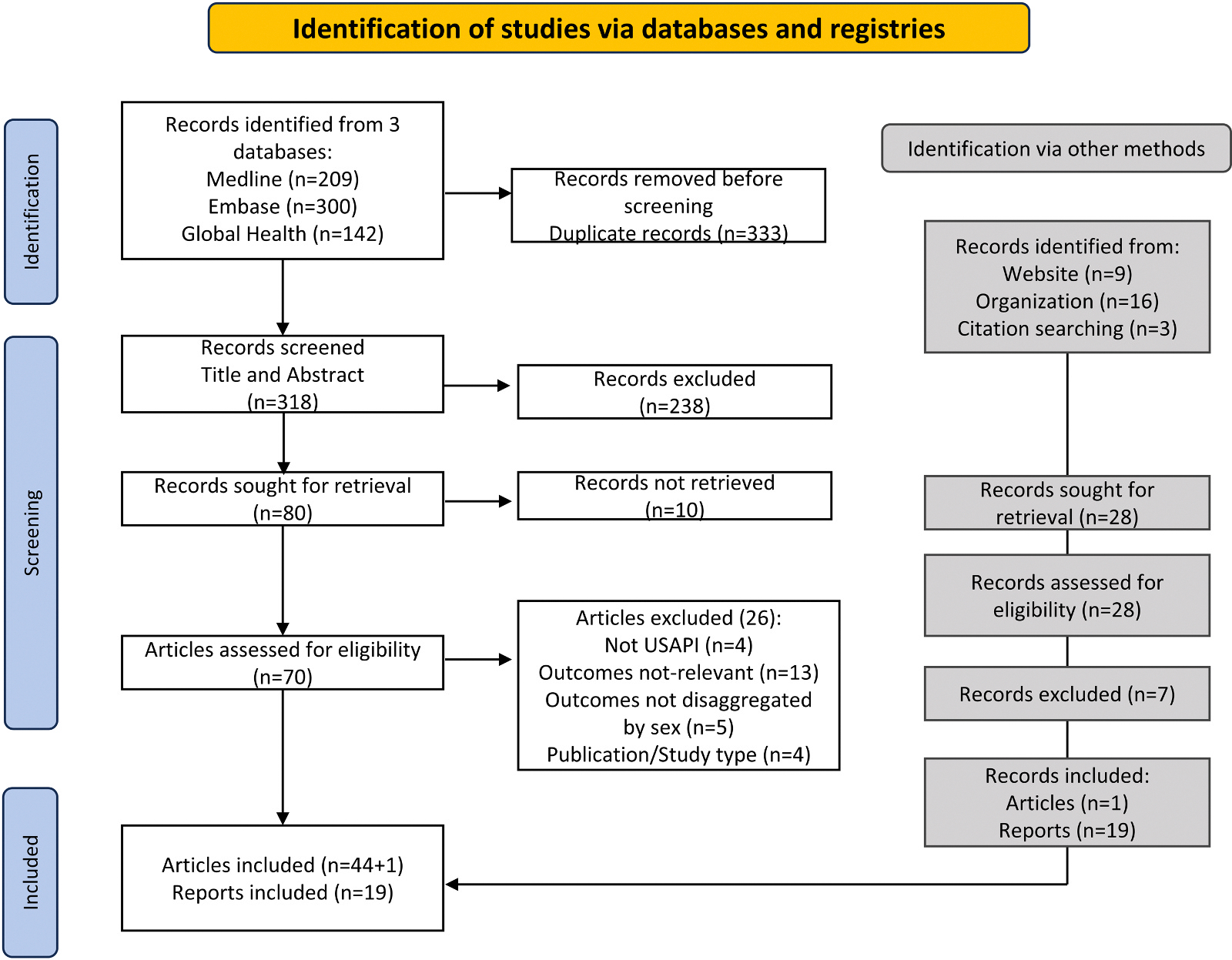
PRISMA Flow Diagram of Study Selection for the prevalence of HPV infection, high-grade precancerous lesions, and correlates and cofactors.

From January 1990 to December 2025, we identified 651 published articles from the 3 databases combined. After removing 333 duplicates and excluding 238 citations (outcomes of interest were not reported, language other than English was used, or the article was an editorial or comment) during the title and abstract screening,10 records that could not be retrieved. Hence, 70 citations were selected for full-text screening. The remaining 44 articles that met the inclusion criteria and provided information on the variables of interest were retained in the review. An additional 1 article and 19 reports were added via citation chaining from identified articles and discussion with experts. For the final review, we included 64 (45 articles and 19 reports) records. A Preferred Reporting Items for Systematic reviews and Meta-Analyses (PRISMA) flowchart [20] for inclusion and exclusion is presented in Fig. 1.

### 3.1. Prevalence of HPV infection and high-grade precancerous lesions among the general female population

Three studies provided estimates of HPV infection or precancerous lesion prevalence among female populations in specific regions in the USAPI (Table 1) [21–23]. The first was a cross-sectional study conducted to assess the prevalence of cervical and anal HPV infection among women attending the obstetric and gynaecologic clinic of the Lyndon B. Johnson Tropical Medical Centre, which provided family planning, prenatal, and gynaecologic services throughout American Samoa during 2008 and 2009 [21]. A sample of 211 women aged 18 years or older, not currently pregnant, at least 6 months postpartum, and having an intact cervix, was included. Cervical specimens for PAP smear and HPV testing were collected for study participants. The polymerase chain reaction (PCR) used with PGMy09/PGMY11 primers were used to detect HPV DNA and HPV genotypes. The study found that among all study participants (with normal and abnormal cytology results) 10% of the cervical samples were infected with an HPV type, in which HPV 6, 16, and 53 were the most common genotypes; half of these infections (5%) were with oncogenic HPV types. Among women with normal cytology results, 8% of cervical samples were found to be HPV positive with an HPV type. The study also found 2% of women had cytological low-grade squamous intraepithelial lesions (LSIL) [21]. (Table 1)

The second study was a randomised controlled trial that aimed to compare HPV DNA detection in self-collected urine samples and in clinician-collected cervical samples in the state of Yap in FSM [22]. The trial recruited 217 women aged 21–65 years from 6 community clinics. Women were included in the study if they were not pregnant, had not had a hysterectomy, and had not been screened in the past 3 years or had abnormal screening results within that period. Study participants were randomized into one of two groups: 1) Clinician-collected cervical sampling followed by self-collected urine sampling; 2) Self-collected urine sampling followed by clinician-collected cervical sampling. For clinician-collected cervical samples, cervical cytology was conducted. Women with abnormal cytology results were followed up with colposcopy and biopsy. Liquid cytology specimens of cervical samples with abnormal cytological results were also tested with reflex HPV testing using the Roche Cobas 4800 PCR system for HPV genotyping. For self-collected urine samples, PCR-based HPV testing was conducted. A small amount of urine specimens was used for cytology. For this review and to be comparable with other included studies, HPV prevalence that was estimated using clinician-collected cervical samples was extracted and presented. HPV DNA prevalence (any type) was 38.3%, with more than half (23.8%) being high-risk HPV. The study also identified women with cytologically abnormal lesions, including 1.4% with LSIL and 1.8% with high-grade squamous intraepithelial lesions (HSIL) [22].

Using data from CDC’s National Breast and Cervical Cancer Early Detection Program (NBCCEDP) from 2007 to 2015, Senkomago and colleagues analysed cervical cancer screening results with screening performed with cytology and human papillomavirus (HPV) testing in four islands (American Samoa, CNMI, Guam, and Palau) [23]. Over the 9-year period, 22,249 cytology (Papanicolaou or Pap) tests were conducted in 14,206 unique women aged 20–64 years. The abnormality rate was 2.4% before 2012 (first screening round), when only the Pap test was used, compared to 1.8% in later years (second screening round), when Pap and HPV DNA co-testing was performed. The rates of LSIL and HSIL were estimated at 1.1%–1.5% and 0.5%–0.6%, respectively (range represents that over 2 screening rounds). There were 2.4–3.8 biopsy-confirmed cases of low-grade cervical intraepithelial neoplasia (CIN1) per 1000 cytology tests, 3.2–4.2 cases of high-grade CIN2 + per 1000 cytology tests, and 0.4–0.6 cases of invasive cervical cancer per 1000 cytology tests [23].

### 3.2. Population exposure to correlates of HPV infection and cofactors for HPV progression to cervical cancer

#### 3.2.1. Correlates of HPV infection

Certain types of sexual behaviour, including age at sexual debut, lifetime number of sexual partners, and sexually transmitted infections (e.g., herpes simplex virus [HSV]–2) are associated with increases in the risk of HPV infection[24]. In the USAPI, data from periodic population surveys have provided an insight into selected correlates to high-risk HPV infections, including age at sexual debut, and lifetime number of sex partners (see Table 2).

The YRBSS has been conducted in five out of six jurisdictions since the 1990s (details of YRBSS rounds for each jurisdiction are presented in the footnotes of Table 2), whereas the RYS has been conducted in FSM’s states since 2016. In both the YRBSS and RYS, male and female high school students in grades 9–12 (equivalent to age 13–18 years) were invited to complete questionnaires anonymously [26,27]. The findings for the percentage of high school girls and boys who reported ever having sexual intercourse, having a sexual debut by age 13 years, and having multiple (more than four) sexual partners are reported in Table 2 and Figure A3, Appendix.

Data from YRBSS revealed a decreasing trend over time in the proportion of students who engaged in sexual behaviours in CNMI (YRBSS 2003–2021) and Guam (YRBSS 1995–2021), with the rate of female respondents reporting having ‘ever had sex’ decreasing significantly from 48.2% to 36.6% (prevalence ratio = 0.8 [95% CI: 0.6–0.9]) in Guam and from 52.1% to 28.2% (prevalence ratio = 0.5 [95%CI: 0.5–0.6]) in CNMI. In Guam, the percentage of female students reported having sexual debut by age 13 years decreased significantly over the survey periods, ranging from 5.9% in the earliest survey to 2.1% in the latest survey (prevalence ratio = 0.4 [95% CI: 0.1–0.9]). Similarly, the percentage of female students reported having more than 4 sexual partners also decreased significantly from 8.9% in the earliest survey to 4.2% in the latest survey (prevalence ratio = 0.5 (95% CI: 0.2–0.9]). This decreasing trend is consistent with the trend observed in the US. (Table 2 and Figure A3, Appendix) In American Samoa (YRBSS 1993–2021) and RMI (YRBSS 2003–2007), the proportions of female and male high school students who reported being engaged in sexual behaviours remain stable over the survey period. In Palau (YRBSS 1999–2021), the percentages of male students reported having sexual debut by age 13 years decreased from 21.0% in 1999–6.4% in 2021 (prevalence ratio = 0.3 [95% CI: 0.2–0.6]) and the percentage having more than 4 sexual partners decreased from 38.3% in 1999–18.1% in 2021 (prevalence ratio = 0.5 [95%CI: 0.3–0.7]). However, no change was observed among female students (Table 2 and Figure A3, Appendix). In RMI, one in three male respondents reported having more than four sexual partners during the survey period. It is of note that YRBSS reports are only available for 2003 and 2007 in RMI (Table 2 and Figure A3, Appendix).

In FSM, the RYS were carried out separately in FSM’s states in different years during 2016–2023, and not all dimensions of sexual behaviour were consistently reported. The overall percentage of female and male students who reported having sexual debut by age 13 ranged from 19.2% (2021) to 23.5% (2016) in Yap, 23.2% (2021) to 33.9% (2019) in Chuuk, and 32.7% (2017) in Pohnpei (Table A4, Appendix).

STI exposure information was gathered from surveillance reports by the CDC’s National Center for HIV, Viral Hepatitis, STD, and Tuberculosis Prevention, detailed in Table A6, Appendix. The chlamydia rates per 100,000 persons in American Samoa and Guam decreased from 209 and 823 in 2018–176 and 640 in 2021, respectively, whereas this rate in CNMI doubled from 311 in 2017–697 in 2021. The gonorrhoeal infection rate per 100,000 persons in CNMI fluctuated between 45 and 66 from 2017 to 2021, while Guam decreases from 176 to 120 from 2017 to 2020[28].

Kosrae, a state in FSM, reported a prevalence of 2.4% for chlamydia and 1.4% for Gonorrhoea in 2017 in 297 adult women [29]. Another state in FSM, Yap, reported a prevalence of 17.5% for any STIs[22]. A modelling study estimated that for women aged 15–49 years in FSM, the prevalence of gonorrhoea, active syphilis, and chlamydia was 1.6%, 1.5%, and 23.9% respectively in 2017, slightly different from 1.7%, 2.7%, and 22.8% respectively in 2000[30].

Across the USAPI, HIV diagnosis in males and females older than 13 years has averaged 5.0/100,000 since 2015[31]. Specific jurisdictional data were limited except for Guam and CNMI. A slight dip in the HIV diagnosis rate in 2020–3.9/100,000 may have been caused by COVID–19 restrictions in accessing health services (Table A6, Appendix). Barriers to accessing sexual health and reproductive health services, especially for young unmarried individuals, such as social stigma and judgmental attitudes imposed by family and community members, including healthcare providers, have been identified based on review of data from other countries in the Pacific regions (Cook Islands, Fiji, and Papua New Guinea)[32]. Although none of these countries are jurisdictions in the USAPI, some of these barriers may be similar in the USAPI.

#### 3.2.2. Cofactors for HPV progression to cervical cancer

##### 3.2.2.1. Cigarette smoking in youth.

Several large-scale surveys report cigarette smoking prevalence in the USAPI. Three surveys reported multi-year estimates of the prevalence of cigarette smoking in youth. The YRBSS was conducted among high school students in grades 9–12, equivalent to students aged 14–18 years in five jurisdictions, except FSM. The RYS was conducted among high school students in grades 9–12 in four states Yap, Chuuk, Pohnpei, and Kosrae of FSM [27,33,34]. The Global Youth Tobacco Survey (GYTS) was conducted in students aged 13–15 years in Guam, FSM and Palau only[35].

YRBSS and RYS defined “current cigarette smoking” as “smoking for at least 1 day during the 30 days before the survey”. According to YRBSS, which reported data disaggregated by sex, a declining trend over the reporting period in the percentages of both female and male students who reported current smoking has been observed in all jurisdictions (Table 2 and Figure A4, Appendix)[25]. Given that a declining trend has been observed, it should be noted that the proportions of young female adolescents who reported current cigarette smoking in the latest YRBSS in some jurisdictions were still approximately four to eight times higher (American Samoa [15.2%, 95% CI: 13.5–17.1], Palau [20%, 17.6–22.7], and RMI [24.4%, 21.4–27.7]), than their counterparts in the US (3.7%, 3.2–4.4). A similar pattern has been observed among male students with the proportions of male students who reported current cigarette smoking in the latest YRBSS were six to 11 times higher than that reported by male students in the US. In FSM, the proportion of current smoking in youth reported by each state separately and showed a stable or increasing trend from 2016 to 2023. The proportions of current cigarette smoking among students in Yap and Pohnpei consistently remained around 30–40%, while these proportions were slightly lower in Chuuk and Kosrae (Table A4, Appendix).

The GYTS defined “current cigarette smoking” as “smoked cigarettes anytime during the past 30 days”, which is slightly different from the YRBSS and RYS. GYTS were conducted in FSM, Guam and Palau. The analysis of GYTS data showed that current cigarette smoking prevalence slightly declined in FSM during 2012–2020 [35]. However, the prevalence of current cigarette smoking remained stable in Guam and increased in Palau during this period [35]. (Table A5, Appendix)

##### 3.2.2.2. Smoking in adults.

Multi-year estimates for adult women were only available for Guam (from BRFSS) to allow trend analysis. BRFSS defines current cigarette smokers as adults who have smoked at least 100 cigarettes in their entire life and who currently smoke, either every day or some days [36]. The survey captured a slow reduction in smoking prevalence among women aged 18–44 years from 25% to 17% during 2011–2019[37]. Other jurisdictions reported less than 15% of women respondents being current smokers in the latest NCD Hybrid Survey Reports[38].

##### 3.2.2.3. Oral contraceptive pills.

High-quality estimates of prevalence of oral contraceptive pill use are not available. However, a Global Burden of Disease modelling study has estimated that oral contraceptive pill use as a proportion of all contraceptive methods has decreased in USAPI jurisdictions since 1970 [39].

### 3.3. Cervical cancer incidence

Cervical cancer incidence rates have been reported in a few publications, with data derived either from the PRCCR, IARC’s reported cancer registration, or GLOBOCAN’s estimates. (Table 3) The latest data over 16 years (2007–2022) from the PRCCR has been published in 2025 [18]. In this cancer registry report, using the case numbers reported from PRCCR, crude rates were calculated by using different population estimates, including the population estimates by the Department of Health (DoH) for each jurisdiction and the United Nations Population Division [18]. With the annual population estimates from the DoH as denominators, the study showed that the overall ASR (age ≥ 20 years) for the USAPI (2007–2022) was 17.2 (95% CI: 15.6–18.8) per 100,000 women (standardised to the US 2000 standard population) (Table 3). The ASR (age ≥ 20) of cervical cancer incidence varies among jurisdictions. RMI had the highest ASR of cervical cancer incidence of 68.9 per 100,000 women (95% CI: 57.3–80.5), followed by FSM with an ASR of 22.6 per 100,000 women. For other jurisdictions, the ASR were 11.8 (95% CI: 7.4–16.2), 9.5 (95% CI: 7.6–11.3), and 8.4 (95% CI: 4.9–12.0) in CNMI, Guam, and American Samoa, respectively [18]. The overall ASR (age ≥ 20 years) of cervical cancer incidence in the USAPI was higher than the national US estimate (ASR (age ≥ 20 years): 10.8 [95% CI: 10.7–10.8] — standardised to US 2000 population)[40]. When applying the United Nations Population Division estimates as denominators, ASR incidence rates for cervical cancer differed slightly (Table 3) [18].

Data from the PRCCR during 2008–2012 was reported in the IARC cancer compendium Volume XI, in which the overall ASR (0–74 years) for cervical cancer incidence in the USAPI was 17.3 per 100,000 women (standardised to Segi world standard population)[41].

GLOBOCAN 2022 also estimated cervical cancer incidence for Guam. The estimated ASR of Guam’s cervical cancer incidence per 100,000 women by the GLOBOCAN 2022 was 19.0 (standardised to Segi World Standard Population) or 26.2 (standardised to the US 2000 Standard Population), whereas the ASR estimated by the PRCCR was 9.5 (7.6–11.3) (standardised to the US 2000 Standard Population). (Table 3) The discrepancy in cervical cancer incidence rates between PRCCR and GLOBOCAN data sources could be attributed to differences in data reporting periods, age groups included, or the GLOBOCAN’s computation method. The GLOBOCAN used population-weighted averages of estimated incidence rates from the US Pacific Islands (2007–2012) and applied them to the 2020 population, rather than using Guam-specific data[42]. Meanwhile, data from PRCCR was recorded through population-based cancer registries in the USAPI.

Among the six jurisdictions, cervical cancer incidence in Guam has been reported via various publications. The Guam Cancer Registry, operational since 1998, started reporting data to the PRCCR in 2007 over different periods [43]. Initial registry data during 1998–2002 reported incidence rates by ethnicities, ranging from 8.4/100,000 women (among Filipinos in Guam) to 27.4/100,000 women (among Micronesians in Guam) (standardized to the US 2000 Standard Population) [43].

Using detailed PRCCR data (2007–2022) and the methodology used in Gopalani et al., 2024[14], alternate source data for annual population estimates (used as denominators) were used for calculation of crude incidence rates, and then alternate standard populations were used to calculate an ASR of cervical cancer (age ≥20 years) standardised to the Segi World Standard Population (for comparison with ASR reported by IARC or GLOBOCAN) and the 2015 World Standard Population (for identifying year to reach the elimination threshold). (See Tables A7.1, A7.2, A7.3, Appendix) When applying the 2022 population estimates from the United Nations Population Division to calculate the crude rate and the US 2000 standard population to calculate ASR incidence rate for cervical cancer in the USAPI (2007–2022) following the method used by Gopalani et al., 2024, the ASR incidence rate was 22.3 (19.1–26.0) per 100,000 women (Table A7.II, Appendix), which is higher than 16.7 (15.1–18.2) per 100,000 women estimated in the PRCCR report. It should be noted that in Gopalani et al., 2024, the ASR incidence rate was calculated for three age groups: 20–39, 40–59, and ≥ 60 years[14], whereas the PRCCR report used twelve 5-year age groups for ages 20 – ≥ 60 years. [18]

#### 3.3.1. Stage distribution of cervical cancer

There are few published data that report cervical cancer cases by stage at diagnosis. According to the PRCCR 20072022 data (Appendix Table A8), a total of 362 cases in the USAPI (74.8%) were diagnosed at stage III or above, 53 (11.0%) cases in stage II, and 69 (14.3%) in stage I (following the International Federation of Gynecology and Obstetrics (FIGO) staging system)[44]. Another study used Tripler Army Medical Center (TAMC) data that captured 214 cervical cancer patients during 1997–2019, and 75 cases were categorized in FIGO stages[45]. The TAMC is located in Hawaii but, in the past, has treated patients from the USAPI through the Pacific Islands Health Care Project[46]. Of 75 cervical cancer patients, 14 (18.7%) were diagnosed at stage IB, 17 (22.7%) at stage IIA-IIB, and 44 (58.7%) at IIIA and above. An older study that also used TAMC data from 2004 to 2014, reported stages III and IV in 62.9% of the 35 cervical cancer patients[47]. Although data are limited, all available sources point towards a late diagnosis of cervical cancer generally, with implications for treatment costs, treatment success, and survival rates. In comparison, among cervical cancers diagnosed in the US during 2018–2022, 41.1% were localized, 36.9% were regional, and 15.7% were distant[48].

### 3.4. Cervical cancer mortality

According to the PRCCR (2007–2022) data, 204 deaths were recorded among 484 patients diagnosed with cervical cancer[19]. The vital registration systems in the six jurisdictions in the USAPI have not consistently used death certificates to confirm causes of death. In most cases, however, verified information from the medical record was used to confirm death from cervical cancer (personal communication, PRCCR representative). Approximately 93% of these reported deaths to the PRCCR occurred within the first 5 years of cervical cancer diagnosis, most of which were diagnosed at late stage, which suggests that the reported deaths were from cervical cancer. There were 81 deaths from RMI, 65 from FSM, 35 from Guam, and 10 from Palau. American Samoa and CNMI recorded 6 and 7 deaths, respectively during the 16 years [19].

Using PRCCR data from 2008 to 2013, van Dyne and colleagues calculated incidence-based mortality (IBM) rates by dividing the number of deaths among incident cases by the population[49]. Cervical cancer IBM age-standardized rates were 34.0/100,000 (18.4–56.0) in RMI, 11.7/100,000 (4.7–24.8) in Palau, 4.2/100,000 (0.6–12.6) in American Samoa, 3.2/100,000 (1.3–6.8) in FSM, and 1.9/100,000 (0.8–3.7) in Guam. (Note that the IBM rate was not calculated for CNMI as the case count was zero).

The only other available mortality data for Guam were from GLOBOCAN 2022, which estimated 13 deaths in 2022 with a mortality ASR rate (all ages, standardized to World Standard Population) of 11.2/100,000 in 2022[50]. GLOBOCAN 2022 estimated the mortality rate for Guam based on published incidence data from the US, and then used PRCCR (2007–2012) survival information[51].

### 3.5. Current cervical cancer prevention activities in the USAPI

#### 3.5.1. Vaccination against HPV

All USAPI jurisdictions introduced HPV vaccines into their national immunization program during 2007–2016[15]. Three territories (American Samoa, CNMI, Guam) receive Vaccines for Children funding. All jurisdictions receive economic assistance, immunization infrastructure support, and limited vaccines through Section 317 of the Public Health Services Act, as well as technical assistance from the CDC[15]. All jurisdictions started with 3-dose quadrivalent vaccine (HPV4), then switched in 2016–2-dose nonavalent vaccine (HPV9), initially including girls aged from 9 to 17 years [52–54]. Among the six jurisdictions, three—American Samoa, Guam, and CNMI — offer HPV vaccination for both girls and boys [54–56]. In terms of vaccine delivery modality, the three freely-associated states (FSM, Palau, and RMI) offered the vaccine almost exclusively through school-based programs, whereas the three US territories have offered it through a mix of school-based vaccination programs and public health clinics[15]. (Table 4)

**Fig. 2.**
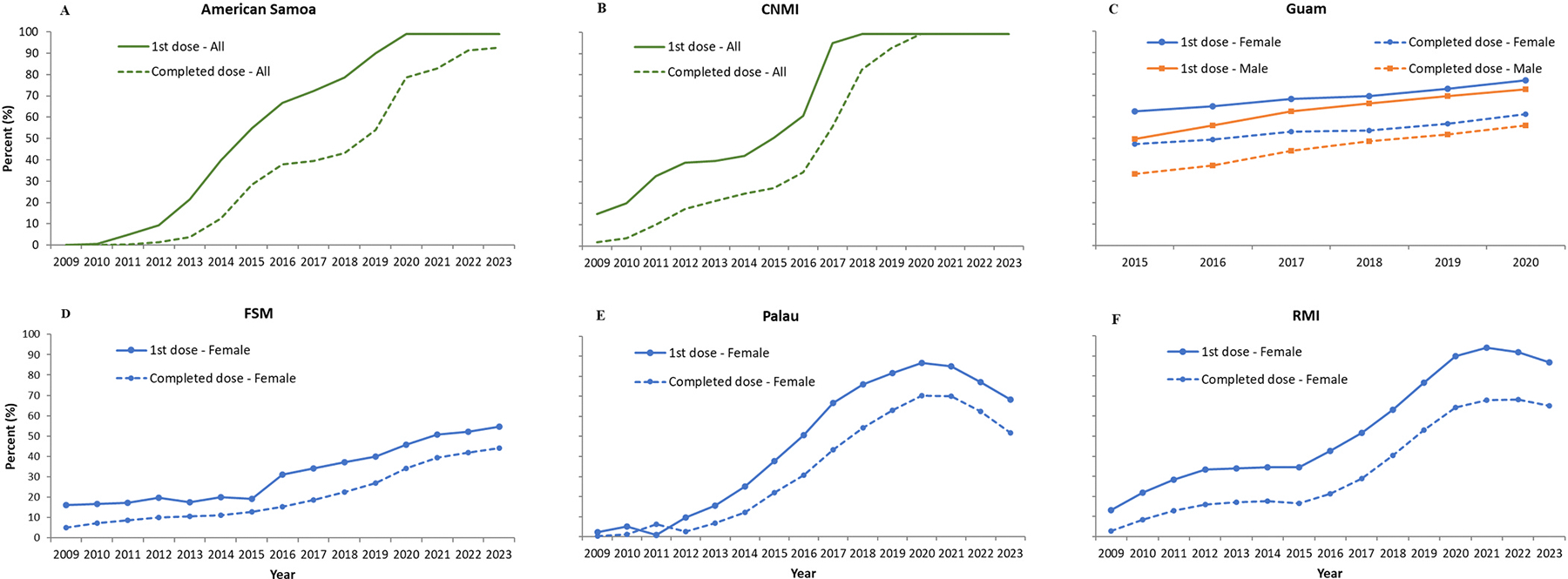
HPV vaccine coverage in the US-Affiliated Pacific Islands. Note: HPV completed dose was defined as receipt of 3 doses of HPV vaccine, or receipt of 2 doses at least 5 months (minus 4 days) apart, before a child’s 15th birthday. Data source: Guam’s HPV vaccination coverage data (coverage data separately presented for females and males) comes from National Immunization Survey-Teen reports. Data for five other jurisdictions come from the Jurisdictional Immunization Information System (IIS), in which the coverage rates represent for females only in FSM, Palau and RMI and for females and males in American Samoa and CNMI. Abbreviations; CNMI: Commonwealth of the Northern Mariana Islands; FSM: Federated State of Micronesia; HPV: Human Papillomavirus RMI: Republic of the Marshall Islands; US: The United States;.

HPV vaccination coverage data for five jurisdictions have been gathered from jurisdictional immunization information systems (IISs) and analysed by the CDC. Data for Guam come from the National Immunization Survey - Teen. (Fig. 2) In addition, HPV vaccine coverage rates were available from the WHO immunization portal for the three freely associated states (FSM, Palau, and RMI) (data not shown)[58]. Data from IISs showed that the coverage of HPV vaccination among females and males has gradually increased since the HPV vaccine program started. American Samoa and CNMI achieved the highest HPV vaccination coverage for both sexes at 92.7% and 99.0%, respectively, in 2023 for completed 2-dose coverage. In Guam, the HPV vaccination completion rate in females and males reached 48.0% and 33.4%, respectively, in 2015 and 61.4% and 56.0%, respectively, in 2020 (Fig. 2). Palau and RMI reached a peak in completed-dose coverage rates for female HPV vaccination at 70.5% and 64.4% in 2020. In 2023, although the HPV vaccination coverage slightly dropped to 51.7% in Palau, this coverage gently increased to 65.1% in RMI. There is an inconsistency in the HPV vaccination coverage data reported between the WHO immunization portal and IIS data for Palau and FSM. In Palau, the reported completed-dose coverage for HPV vaccination after the COVID pandemic remained high at 51.7%–70.2% in the IIS data but decreased to 21.0%–40.0% in the WHO immunization data. In FSM, the completed HPV vaccine coverage rates as reported by the IIS data gradually increased over time since the program started and reached a peak coverage of 44.2% in 2023; however, rates were higher on the basis of WHO immunization data. These disparities in the coverage rates between IISs and the WHO immunization portal could be potentially due to the differences in the methods used in calculating the coverage rates [15,58].

#### 3.5.2. Cervical cancer screening

##### 3.5.2.1. Up-to-date cervical cancer screening status in the region.

Table 5 summarizes cervical cancer screening practices in the six USAPI jurisdictions. Over the past few decades, cervical cancer screening in the USAPI has been supported by the US through several programs, including CDC’s NBCCEDP, Title X Family Planning for all six jurisdictions, the HRSA-administered Title V Maternal Child Health program, and the FQHCs for American Samoa, CNMI, Guam, and Palau. Since 1998, four of the six jurisdictions received funding from NBCCEDP, including American Samoa (1998–present), Guam (2002–present), Palau (1998–present), and CNMI (1998–2002, 2007–present)[23].

Since 2018, the NBCCEDP has been operational in RMI[73]. Each jurisdiction recommended specific cervical cancer screening and management pathways, based on their political and socioeconomic conditions. At present, for four jurisdictions that are supported by NBCCEDP, cervical cancer screening programs follow screening recommendations from the U.S. Preventive Services Task Force (USPSTF), in which women aged 21–65 years are recommended for screening every 3 years using a cytology test or every 5 years if an HPV test and a cytology test are used as co-testing for women aged 30–65 years[61]. The American Society for Colposcopy and Cervical Pathology (ASCCP) guidelines are recommended for follow-up of women who have abnormal screening results [62]. Two jurisdictions, FSM and RMI, developed their own cervical cancer screening standards in 2009 and 2010, respectively [70,74]. According to the FSM standard of practice for cervical cancer early detection recommendations, screening with visual inspection of the cervix after acetic acid application (VIA) at least twice in a lifetime for women aged 25–45 years was recommended for settings with a core resource level. For settings at an expanded resource level, 5-year screening and treatment for women aged 25–45 years and 3-year cytology screening for women aged 21–65 years were recommended [74]. Since 2020, one of four states in FSM has been supported by the Pacific Against Cervical Cancer (PACe) project to improve the cervical cancer screening program using HPV tests[75]. The experience from the PACe project will help to guide the scaling up of cervical cancer screening by using primary HPV screening in the FSM in the future. For RMI, in the 2012 Cancer Control Plan, both VIA and cytology were recommended for cervical cancer screening. However, given the “widely recognized historical and unresolved challenges with cytology screening in RMI setting,” the National Comprehensive Cancer Control Plan (2017–2022) set a strategy for cervical cancer screening using VIA tests as a core screening method[76]. This Cancer Plan was developed before the most recent WHO cervical cancer screening guidelines in 2021, in which high-performance screening technologies, such as HPV testing, has been recommended[77]. Nevertheless, as RMI has been registered in NBCCEDP since 2018, women who are eligible for this program will receive cytology screening and follow the cervical cancer screening management as recommended in the US[73].

Over the last few decades, cytology-based cervical cancer screening has remained the principal screening technique in the USAPI, despite the diversities and limitations in political, geographic, and socioeconomic conditions among different jurisdictions in this region[78]. Cytology-based screening services have faced many challenges in terms of workforce, laboratory infrastructure, transportation, service delivery and accessibility in the USAPI, which has affected the effectiveness and sustainability of cytology-based screening in this region. For example, in Guam, cervical samples are sent to Hawaii for cytology testing. Other USAPI jurisdictions also send their samples off-island for testing[13]. Consequently, it can take between a week and a month for women to receive their results, and costs are higher because of additional shipping cost. In addition, the long waiting time results in delays in diagnosis and treatment. Cytology screening also requires multiple visits for screening, result collection, and precancerous treatment, as needed. Inadequate transportation and communication, coupled with multiple steps, results in a high loss to follow-up and delayed diagnosis and treatment[78]. Cytology-based screening is labour-intensive, expensive, and inefficient in USAPI[78]. In addition to technical issues with cytology screening, several barriers and factors, including the availability of resources and facilities, trust in the health care system, culture and tradition, socioeconomic status, and awareness, have been identified that hinder women from participating in screening, as well as contribute to late presentation for treatment among the Pacific island countries[79].

##### 3.5.2.2. Screening coverage.

Table 5 provides an overview of the cervical cancer screening coverage in the six jurisdictions in the USAPI region, based on two main data sources: population surveys (e.g., BRFSS [Guam], NCD Hybrid surveys [AM, CNMI, Palau, RMI, two states of FSM — Kosrae and Pohnpei]) and program data from the HRSA FQHC reports. It is of note that cervical cancer screening data from BRFSS or NCD hybrid surveys were collected via questionnaires (via phones and cell phones in BRFSS or face-to-face in NCD hybrid surveys) asking about whether they received a screening test and how long it has been since their last screening test [36,80]. At the time of our review, BRFSS data were only available up to 2020, and only cervical cancer screening with cytology was reported. Similarly, as NCD Hybrid surveys have been conducted since 2016–2018, cervical cancer screening rates were reported for cytology only in American Samoa, CNMI and Palau. In RMI and FSM, reported cervical cancer screening rates included either VIA or cytology. Cervical cancer screening coverage estimated from HRSA-funded FQHC databases based on medical records of women who received cervical cancer screening tests (cytology tests for those aged 23–65 years or HPV tests for those aged 30–65 years) among those aged 23–65 years with a visit during the measurement period[81].

Although the FQHC provided data annually from 2018 to 2022 as presented in Table 5, we used data reported from years before the COVID–19 pandemic for comparison purposes. Among six jurisdictions, Guam’s 2018 BRFSS results stand out for consistently the highest screening coverage rate for women who received cytology in the past 3 years (67.5% [95% CI: 59.7–75.2])[65]. The cervical cancer screening coverage rates that were recorded in the HRSA databases have fluctuated over years with the lowest rate being 40% in 2018 and the highest being 65.7% in 2020 [81]. (Table 5) It should be noted that the screening rate reported by BRFSS was reported based on responses by survey participants and could be affected by recall bias while the rates reported by HRSA were the actual numbers of women who received screening. These might explain variations in screening rates between two data sources. Despite high screening coverage compared to other USAPI, screening coverage in Guam was lower compared to the US territories of Puerto Rico and US Virgin Islands and compared to the US mainland (50 states and DC)[17].

The cervical cancer screening coverage reported in Palau ranked second after Guam. In Palau, the NCD Hybrid Survey 2017 reported that 60.3% of women aged 21–65 years has cytology within the past 3 years [69]. In Palau, cervical cancer screening coverage for women of screening age (23–65 years) in HRSA-funded FQHCs was slightly lower at 49.0% in 2018, 44.9% in 2019, 36.7% in 2020, 38.5% in 2021, and 36.9% in 2022[81]. (Table 5)

Cervical cancer screening coverage in CNMI ranked third with 43.2% of women who responded to the 2016 Hybrid survey having received a screening test in the past 3 years[64]. In HRSA program data, although data for 2016, 2017, or 2018 were not available for CNMI, cervical cancer screening coverage was 14.2% in 2019, and increased to 35.7% in 2020 and 43.0% in 2021, even with the impacts of the COVID–19 pandemic[81]. Among the four jurisdictions that received funding and support from NBCCEDP and HRSA’s FQHCs, the screening uptake in American Samoa was the lowest; 29.3% of women reported receiving a cervical cancer screening test in the past 3 years in the 2018 Adult Hybrid survey[63]. Two jurisdictions, RMI and FSM, recommended either cytology or VIA screening. In RMI, 33.6% of women responded in the 2018 Hybrid survey that they received a cervical cancer screening test in the past 3 years, and 54.2% reported never having received cervical cancer screening[63]. Cervical cancer screening coverage in the HRSA’s program data was consistently lower than 30% during 2018–2022 (Table 5).

In FSM, two hybrid surveys were conducted separately in two states of FSM (Pohnpei and Kosrae) in 2019 only. The prevalence of women aged 21–65 years who self-reported having a screening test over the past 3 years was 28.1% in Pohnpei [67] and 38.5% in Kosrae [29]. Cervical cancer screening uptake among women aged 18–69 years in Chuuk was relatively low at 13.1%, as reported in the WHO STEPwise approach to NCD risk factor surveillance (STEPS) in 2016[68]. Although there was no overall cervical cancer screening uptake reported for FSM through population-based surveys, program data from HRSA suggested fluctuation, from 41.4% to 14.6% during 2018–2022. (Table 5) Overall, compared to the cervical cancer screening coverage in the mainland US (~80.2%), the coverage in the USAPI was substantially and consistently lower.

##### 3.5.2.3. Cervical precancer and cancer treatment status in the USAPI.

Information on cancer treatment status in the USAPI was derived from a report on cancer in the USAPI (2007–2022). According to this report, all jurisdictions in the USAPI have capacity to treat precancerous lesions and stage 1 A of cervical cancer, given the availability of general surgeons and obstetrician-gynecologists as summarized in Table 6. Beside building capacity on cervical cancer screening, the PACe project is also working on expanding the access to precancer treatment in Yap state of FSM with the introduction of thermal ablation and in Guam with colposcopy and LEEP[75]. Cancer treatment capacity for later stages (II+) has been extremely limited in most island jurisdictions [18]. Guam has the most capacity for cancer treatment with general surgery, radiotherapy, and chemotherapy available. Brachytherapy and hormonal therapy are available but limited. CNMI has one oncologist but no radiotherapist, which these explain for the availability of surgery and chemotherapy, but not radiotherapy. FSM, Palau, and RMI only have general surgeons. All jurisdictions have some forms of budget earmarked for off-island treatment, most often in the Philippines because of costs. However, budgets are constrained and limited, and overseas treatment referrals are generally limited to those who are diagnosed early and to whom physicians have determined a 5-year survival rate of more than 50.0% (Table 6)[18].

## 4. Discussion

This review extended the scope and updated a previous review of cervical cancer incidence and mortality in the Pacific region[82]. We assessed the most up-to-date available information on the prevalence of cervical HPV infection and HSIL, cervical cancer incidence and mortality, and factors associated with HPV infection and progression to cervical cancer, as well as providing a snapshot of the status of prevention and control towards cervical cancer elimination in the USAPI. Findings from this study can be used to inform governments, policy makers, and regional and international agencies who aim to support the USAPI to achieve cervical cancer elimination.

### 4.1. Summary of findings and comparison with existing literature

Data were scarce on HPV infection and precancerous lesions in the USAPI. This could be a focus for future research. Only two population-based cross-sectional studies have been conducted to estimate HPV prevalence among women in American Samoa and Yap and one study analysed data from NBCCEDP. From the limited data available, HPV16 appeared to be the most prevalent genotype among women in the general population in the region [21,47]. The HSIL prevalence estimated from the NBCCEDP data (with more than 20,000 Pap smears tested in 14,200 women during 2007–2015 in 4 jurisdictions of the USAPI) was 0.5%–0.6% [23], which is relatively low compared to other Pacific Islands. For example, in Papua New Guinea (PNG), the HSIL prevalence was 6.2%, as calculated among 4285 women who participated in a large-scale, prospective, single-arm HPV screen-and-treat trial in PNG [83]. The difference between HSIL prevalence in these settings could possibly be caused by the cervical cancer screening status in each setting. In the USAPI, NBCCEDP and possibly other screening initiatives have been operating in these jurisdictions over the past few decades, so some women will have been screened several times and treated for abnormal lesions.

The available data from YRBSS showed some similarities to the mainland US in terms of the proportion of adolescents reporting engaging in sexual behaviour and cigarette smoking in CNMI and Guam. However, exposure to these risk factors was higher in American Samoa, Palau, and RMI, compared to the mainland US. A point should be noted that whereas the participants in the YRBSS were high school students aged 13–18 years old, questions regarding sexual activities, including new partners or sexual relationship with a partner who has had multiple partners were not included in the questionnaire. Therefore, the actual number of sexual partners may be underreported. Rates of reported chlamydia cases in Guam and CNMI were close to the 2021 rate in the US of 628 per 100,000 women[84]. STIs, such as chlamydia and gonorrhoea, can be managed according to symptoms when laboratory tests are not available in a country[85]. However, sexual and reproductive health services in the Pacific Islands are reported to be inaccessible, inconvenient, or not culturally sensitive[32]. Considering the limited access and utilisation of STI and sexual health services coupled with unascertained sexual behaviour patterns[32], enhanced and tailored efforts, such as public health outreach and school-based education curricula, may help prevent STI infections in the USAPI.

Based on the latest PRCCR data during 2007–2022, the overall ASR of cervical cancer incidence per 100,000 was 17.2 (15.6–18.8) in the USAPI [18]. RMI recorded the highest rate of 68.9 (57.3–80.5), and the lowest rate was observed in American Samoa at 8.4 (4.9–12.0), standardized to the US 2000 Standard Population. This variation in cervical cancer rates among jurisdictions persists when alternative population denominator estimates were used. Additionally, it is noted that ASR incidence rates for cervical cancer in the USAPI also varied, depending on the population estimates used to calculate the crude incidence rates, on the standard populations used to calculate age-standardized rates, and also on how ages were grouped together.

The completed two-dose coverage rates of HPV vaccination over time in all jurisdictions were higher than the global average [8], and coverage has reached the HPV vaccination target for cervical cancer elimination (90%) in American Samoa and CNMI. This success has been achieved within 15 years of introducing HPV vaccine into their national immunization programme with support from the US government. It is notable that now many countries have moved to one-dose vaccination, following a major WHO review in 2022[86]. One-dose vaccination should be an enabler of high and sustained coverage in many settings, and will be an important future consideration in the USAPI.

However, cervical cancer screening coverage exhibited significant variation across the USAPI, ranging from approximately 30% in American Samoa, RMI, and FSM, to around 50% in Guam and Palau. This divergence underscores the heterogeneity in healthcare access and the quality of health services across the different jurisdictions. Factors contributing to low screening coverage include the availability and quality of healthcare services, accessibility, education, and cultural and religious beliefs, which have been identified as barriers to undergoing screening tests in the Pacific Island countries [79,87].

USAPI jurisdictions could consider the role of WHO guidelines for ‘screen and treat’, which has been implemented successfully in some settings, such as Papua New Guinea, where a point-of-care ‘screen and treat’ intervention has proven successful[83]. This screening management approach, currently being piloted in Yap, involves HPV testing, followed by treatment with thermal ablation, if eligible[75]. In addition, having a self-collection option, which is being pilot-tested in Yap, may increase the screening uptake by allowing women to take samples at home or at healthcare facilities.

Access to cancer treatment is currently very challenging in the USAPI, and most patients need to seek care off-island, including those with cancer diagnosed at a late stage in Guam, with resulting high costs and treatment delays. In the past, the shortage of surgeons and anaesthesiologists has led to the establishment of organised postgraduate surgical training initiatives in Pacific Island countries[88]. One such initiative — the Pacific Island Projects — was funded by AusAID and has been a successful partnership in delivering and building medical services in Fiji, PNG, and Timor Leste [89,90]. This has helped in providing comparatively low-cost surgical services in PNG, Fiji, and neighbouring islands. A 2-year gynaecologic oncology training fellowship program, designed by the International Gynaecologic Cancer Society (IGCS), can be an option to build capacity for cervical cancer treatment in low-resource settings[91]. Such coordinated actions towards in-island capacity development could be considered by the USAPI and implemented through the Pacific Regional Comprehensive Cancer Control Programme and Partners.

### 4.2. Limitations and strengths

This study has several limitations. Firstly, we were not able to estimate the age-standardised incidence of cervical cancer adjusted for the impact of the benign hysterectomy rate, given the paucity of hysterectomy data in the USAPI. The ASR incidence would be higher than current estimates, given that the denominator of the susceptible female population would be smaller after adjustment for the number of women who have experienced hysterectomy. We were also not able to estimate the mortality of cervical cancer because the causes of death were not uniformly confirmed by death certification. These limitations are common in developing countries and other Pacific Islands, where death certificates are not always coded to ICD–10, or where full death certification has not been a requirement in all jurisdictions[92]. [93], With the unique conditions in the USAPI, the cancer registry in the USAPI has faced many challenges in collecting cervical cancer cases, particularly in identifying deaths caused by cancer. Recall bias may arise in the screening coverages reported by the BRFSS because the survey asked women whether they received a screening test during the past 3 years (for cytology screening) or 5 years (for HPV screening). Similarly, a selection bias may arise in the screening coverages estimated from the HRSA database due to differential access to healthcare services, particularly between urban and rural (i.e., small adjacent islands) populations in each jurisdiction. Women with better proximity to clinics or higher health literacy may be overrepresented in the data, potentially inflating estimates of screening and vaccination coverage. Lastly, it should be noted from this review that there is little published literature on the treatment of precancerous lesions and invasive cervical cancer in the USAPI. The collected information mainly comes from local government program reports, discussions with local partners, and the experience of the PACe project. A part from enormous efforts on HPV vaccination and cervical cancer screening, improvement of treatment capacity for cervical precancer and cancer will be an area of focus for governments and organizations in the USAPI.

In terms of review process, to ensure various themes of the research question are fully addressed, a structured narrative review with theme-specific review approaches was used. We included all publications that met the inclusion and exclusion criteria. Some of these publications could have some publication bias. For example, only two studies on HPV prevalence were conducted in American Samoa and in Yap state in FSM. The findings from these two studies may not be representative for other jurisdictions in the USAPI; Additionally, the studies’ sample sizes were small in several cases limiting the generalizability of the findings for regional population. Due to limited numbers of publications identified through literature search, we presented all the identified literature and additionally included grey literature to be as comprehensive as possible.

This study has many strengths. This is a structured narrative review, which comprehensively covered a full range of topics of cervical cancer burden, its risk factors, and overview of cervical cancer prevention and control status in the USAPI region. This review provides the most up-to-date evidence for policy makers and health professionals who are considering any interventions on cervical cancer in this region. The study has arisen from our previous experience of reviewing the burden of cervical cancer in other settings [94,95]. We reviewed published literature, online databases, survey reports, health system registries, and grey reports from six jurisdictions for relevant data. We were able to gather up-to-date available data on cervical cancer incidence, mortality, and its risk factors in the USAPI. Using the latest health information registration systems data, we also provided an updated snapshot of the most current status of HPV vaccination, cervical cancer screening, and treatment in the region.

### 4.3. Implications for policy, services, and research

Findings from this review highlight that the burden of cervical cancer in the USAPI is substantially higher than that reported for the mainland US. However, data on the HPV prevalence and precancerous lesions, as well as reliable cervical cancer incidence and mortality data, are limited for this region. Despite the uncertainties, additional implementation research could support cervical cancer prevention efforts in the USAPI.

Current evidence highlights numerous challenges in cervical cancer screening and treatment, contributing to delays in diagnosis and care. Barriers to screening, such as limited resources and facilities, insufficient follow-up systems, cultural factors, low awareness, and socioeconomic constraints, have been identified in previous research[88]. Jurisdictions could consider these factors when designing effective screening programs. In addition, reliance on cytology-based screening, which depends on overseas laboratories in all USAPI jurisdictions, suggests that point-of-care HPV testing may be beneficial. On-site testing could reduce delays and improve access to timely care in the geographically unique USAPI.

Self-collection for HPV testing, recently approved in the US, offers another promising strategy to expand screening coverage by providing women with more accessible options. Initiatives, such as the PACe project currently underway in Guam and Yap, aim to build cervical cancer screening capacity in the region[75]. Addressing treatment gaps, particularly for cervical precancer and cancer, requires enhanced training and equipment to strengthen healthcare capacity. Insights from an ongoing initiative to expand cancer treatment capacity in Indo-Pacific countries could guide similar efforts in the USAPI[90].

Beyond specific cervical cancer prevention programs, the USAPI may benefit from strengthened health service access and civil registration systems, such as death registration. Health services could be improved by strengthening the six health system building blocks, including: leadership and governance, service delivery, health workforce, health information systems, access to medicines and technologies, and financing[96]. For example, in terms of service delivery of cervical cancer screening, ensuring patient-centred, timely, and efficient approach by setting up point-of-care settings for HPV screening may help to minimize the loss-to-follow-up in the USAPI. Similarly, civil registration systems can be improved by ensuring the completion of death certificates for all deaths in the community and at health facilities, thereby improving the quality of the PRCCR. Improving the quality of the PRCCR mortality data is integral to enhancing cancer data collection and analysis, ultimately supporting the planning and implementation of effective cancer control interventions in these jurisdictions.

Going forward the experience from the PACe project and its increasing alignment with a major Indo-Pacific initiative EPICC (Elimination Partnership Cervical Cancer), funded by the Australian government and the Minderoo Foundation, will be an important enabler of regional coordination towards supporting country leadership for sustainable elimination programs.

## 5. Conclusions

Current cervical cancer incidence in the USAPI is high overall with considerable variation among USAPI jurisdictions and is substantially higher than in the US, compounded by late-stage diagnoses and limited treatment capacity. HPV vaccination programs began 17 years ago, in which two jurisdictions have achieved 90% coverage. However, implementation of effective cervical cancer screening and precancer and cancer treatment has faced many challenges due to geographic isolation, limited infrastructure, and workforce shortages. Findings from this study suggest the USAPI could benefit from strengthening cervical cancer screening, and precancerous lesion and invasive cervical cancer treatment. Initiatives like the PACe project are actively addressing these gaps and building capacity to advance toward the goal of cervical cancer elimination in the USAPI.

## Supplementary Material

Appendix A. Supplementary material

## Figures and Tables

**Table 1:** Prevalence of HPV infection and high-grade precancerous lesions among the general female population in the USAPI.

Article	Sample population	Age (years)	Sample size	Prevalence of any type HPV infection (high risk HPV)		Cytological HSIL
				All participants (normal and abnormal cytology)	Participants with normal cytology	
						
American Samoa						

Hernandez, Ka’opua [21]	Women not currently pregnant and at least 6 months postpartum, and no prior hysterectomy	18–82	211 (53.0% ever screened with Pap smear)	10.0% (5.0%)	8.0% (NA)	HSIL: not detected LSIL: 2.0%
**FSM**						
Hernandez, Tareg [22]	Women not currently pregnant and at least 6 months postpartum, and no prior hysterectomy, had not been screened in the past 3 years or had abnormal screening results within the past 3 years	21–65	217 (60.0% ever screened with Pap smear or VIA)	38.3% (23.8%)	23.5% (15.1%)^α^	HSIL: 1.8% LSIL: 1.4%
**American Samoa, CNMI, Guam, Palau**						
	Women served by the NBCCEDP in the four USAPI jurisdictions from 2007 to 2015	21–65	14,206*	NA		HSIL: 0.5–0.6%# LSIL: 1.1–1.5%#

Abbreviations: NBCCEDP: National Breast and Cervical Cancer Early Detection Program; Low-grade Squamous Intraepithelial Lesion (LSIL); High-grade Squamous Intraepithelial Lesion (HSIL); FSM: Federated States of Micronesia; CNMI: Commonwealth of the Northern Mariana Islands; HPV: Human Papillomavirus; Pap smear: The Papanicolaou test; NA: Not available.

(α) any type HPV prevalence and high-rick HPV prevalence rates among the normal cytology women were calculated based on data shared by the study team at the University of Hawai’i.

(*) The screening data were collected in two screening rounds. The first screening round: 10,677 unduplicated women screened, 10,933 Pap tests conducted; The subsequent screening rounds: 3529 unduplicated women screened, 11,316 Pap tests conducted[23].

(#) Range represents the rates estimated over two screening rounds.

**Table 2:** Trends of sexual behaviour and cigarette smoking among youths in the US-Affiliated Pacific Islands.

Outcomes by jurisdiction^a^	Female			Male			Prevalence ratio (95% Confidence Interval) between males and females^c^	
		
	Earliest survey prevalence % (95% CI)	Latest survey prevalence % (95% CI)	Prevalence ratio (95% CI)^b^	Earliest survey prevalence % (95% CI)	Latest survey prevalence % (95% CI)	Prevalence ratio 95% CI)^b^	Earliest survey	Latest survey

**Ever had sex**								
American Samoa	26.7(21.7–32.4)	27.2(24.9–29.5)	*1.0(0.9–1.2)*	56.9(50.4–63.1)	44.2(41.3–47.2)	*0.8(0.7–0.8)* *	*2.1(1.8–2.6)* *	*1.6(1.5–1.8)* *
CNMI	52.1(49.0–55.1)	28.2(25.7–30.8)	*0.5(0.5– 0.6)* *	55.9(52.7–59.0)	28.9(26.4–31.6)	*0.5(0.5–0.6)* *	*1.1(1.0–1.2)*	*1.0(0.9–1.2)*
Guam	48.2(39.4–57.1)	36.6(30.2–43.5)	*0.8(0.6– 0.9)* *	44.6(36.5–53.0)	37.7(32.3–43.4)	*0.9(0.7–1.0)*	*0.9(0.7–1.2)*	*1.0(0.9–1.2)*
Palau	26.0(22.8–29.4)	29.2(26.2–32.5)	*1.1(0.8–1.5)*	71.0(66.8–74.9)	36.0(32.4–39.8)	*0.5(0.4–0.6)* *	*2.7(2.2–3.5)* *	*1.2(0.9–1.7)*
RMI	46.3(42.7–49.9)	47.0(42.9–51.2)	*1.0(0.9–1.2)*	73.5(70–76.8)	72.0(68.0–75.8)	*1.0(0.9–1.1)*	*1.6(1.4–1.9)* *	*1.5(1.4–1.7)* *
US	47.7(43.5–51.9)	30.6(28.3–33)	*0.6(0.6– 0.7)* *	52.2(48–56.2)	29.3(26.9–31.8)	*0.6(0.5–0.6)* *	*1.1(1.1–1.1)* *	*1.0(0.9–1.0)*
***Sexual debut by age 13 years***								
American Samoa	3.1(1.8–5.3)	4.0(3.1–5.1)	*1.3(0.7–2.3)*	15.7(12.6–19.3)	13.0(11.1–15.1)	*0.8(0.6–1.1)*	*5.1(2.8–9.0)* *	*3.3(2.4–4.5)* *
CNMI	7.6(6.1–9.4)	4.2(3.2–5.5)	*0.6(0.4– 0.8)* *	15.9(13.7–18.3)	4.0(3–5.3)	*0.3(0.2–0.4)* *	*2.1(1.6–2.7)* *	*1.0(0.6–1.5)*
Guam	5.9(2.4–13.7)	2.1(0.9–4.7)	*0.4(0.1–0.9)* *	11.4(7.1–17.8)	6.1(3.7–10.0)	*0.5(0.3–0.9)* *	*1.9(0.9–4.4)*	*2.9(1.5–5.7)* *
Palau	1.7(0.9–3.0)	2.6(1.6–4.0)	*1.5(0.4–5.7)*	21.0(17.6–24.8)	6.4(4.7–8.5)	*0.3(0.2–0.6)* *	*12.4(4.6–32.9)* *	*2.5(0.9–7.1)*
RMI	3.7(2.5–5.5)	3.0(1.8–4.9)	*0.8(0.4–1.8)*	18.7(15.7–22.1)	14.3(11.5–17.6)	*0.8(0.5–1.2)*	*5.1(2.5–10.2)* *	*4.8(2.6–8.9)* *
US	4.4(3.5–5.6)	3.1(2.6–3.7)	*0.7(0.6–0.8)* *	12.2(10.6–14.0)	3.2(2.4–4.0)	*0.3(0.2–0.3)* *	*2.8(2.5–3.1)* *	*1.0(0.9–1.2)*
***Having more than four sexual partners***								
American Samoa	3.3(1.7–6.3)	6.5(5.3–8.0)	*2.0(1.1–3.4)*	20.9(16.4–26.3)	20.0(17.7–22.5)	*1.0(0.8–1.2)*	*6.3(3.7–11.0)* *	*3.1(2.4–3.9)* *
CNMI	11.8(9.8–14.0)	4.0(3.0–5.4)	*0.3(0.2–0.5)* *	18.5(16.1–21.1)	6.0(4.7–7.5)	*0.3(0.2–0.4)* *	*1.6(1.3–2.0)* *	*1.5(1.0–2.2)* *
Guam	8.9(4.3–17.4)	4.2(2.7–6.6)	*0.5(0.2–0.9)* *	14.8(8.9–23.4)	7.9(5.5–11.4)	*0.5(0.3–0.9)* *	*1.7(0.9–3.3)*	*1.9(1.1–3.1)* *
Palau	4.5(3.2–6.4)	2.9(1.9–4.3)	*0.6(0.2–1.8)*	38.3(34.1–42.8)	18.1(15.3–21.3)	*0.5(0.3–0.7)* *	*8.5(4.6–15.8)* *	*6.2(2.5–15.6)* *
RMI	12.8(10.6–15.4)	10.4(8.1–13.1)	*0.8(0.5–1.2)*	37.7(33.7–41.8)	29.0(25.1–33.1)	*0.8(0.6–1.0)* *	*3.0(2.1–4.2)* *	*2.8(2.0–3.8)* *
US	13.1(11.0–15.5)	5.1(4.3–6.0)	*0.4(0.4–0.4)* *	19.3(15.8–23.3)	6.8(5.8–8.0)	*0.4(0.3–0.4)* *	*1.5(1.4–1.6)* *	*1.3(1.2–1.5)* *
***Current cigarette smoking (in the past 30 days)***								
American Samoa	37.1(31.9–42.6)	15.2(13.5–17.1)	*0.4(0.3–0.5)* *	40.4(33.6–47.6)	23.0(20.9–25.2)	*0.6(0.5–0.7)* *	*1.1(0.9–1.3)*	*1.5(1.3–1.8)* *
CNMI	51.2(48.0–54.3)	5.2(4.1–6.7)	*0.1(0.1–0.1)* *	46.4(43.3–49.6)	8.6(7.2–10.3)	*0.2(0.2–0.2)* *	*0.9(0.8–1.0)*	*1.7(1.2–2.3)* *
Guam	43.3(35.7–51.3)	7.7(5.3–11.1)	*0.2(0.1–0.3)* *	39.0(31.6–46.9)	15.6(11.9–20.1)	*0.4(0.3–0.5)* *	*0.9(0.7–1.2)*	*2.0(1.4–2.8)* *
Palau	29.8(27.4–32.3)	20.0(17.6–22.7)	*0.7(0.5–0.9)* *	48.0(45.1–50.8)	27.8(24.8–31.0)	*0.6(0.4–0.8)* *	*1.6(1.3–2.0)* *	*1.4(1.0–2.0)*
RMI	25.2(22.4–28.1)	24.4(21.4–27.7)	*1.0(0.8–1.2)*	51.7(48.1–55.3)	40.9(37.3–44.7)	*0.8(0.7–0.9)* *	*2.1(1.7–2.6)* *	*1.7(1.4–2.0)* *
US	34.9(32.3–37.7)	3.7(3.2–4.4)	*0.1(0.1–0.1)* *	34.7(31.8–37.7)	3.9(3.1–4.9)	*0.1(0.1–0.1)* *	*1.0(1.0–1.0)*	*1.1(0.9–1.2)*

Data Source: Youth Risk Behaviour Surveillance Systems (YRBSS) [25]

Abbreviations: CNMI: Commonwealth of the Northern Mariana Islands; RMI: Republic of the Marshall Islands; US: The United States

a YRBSS data are available for different years in each jurisdiction: American Samoa: 1993–2021; CNMI: 2003–2021; Guam: 1995–2021; Palau: 1999–2021; RMI: 2003–2007; US: 1999–2021.

b Prevalence ratio was estimated based on the prevalence of each outcome in the latest versus the earliest YRBSS survey, calculated separately for females and males

c Prevalence ratio was estimated based on the prevalence of each outcome in males versus females, calculated separately for the earliest and the latest YRBSS survey.

* Significant change in the prevalence over time with P < 0.05;

**Table 3:** Age-standardized incidence rates of cervical cancer in the US-Affiliated Pacific Islands.

US-Affiliated Pacific Islands	PRCCR (2007–2022) [18]			GLOBOCAN 2022[1]				IARC Vol. XI (2008–2012)[41]		
				
	Cases	Crude rate	ASR* incidence rate (age 20+, 95% CI)	Cases	Crude rate	ASR incidence (0–85+) (α)	ASR incidence (age 0–85+) (β)	Cases	Crude rate	ASR Incidence (age 0–74) (∞)

USAPI - Overall 2007— 2022	484	23.5^a^	17.2 (15.6–18.8)^a^ 16.7 (15.1–18.2)^b^	NA	NA	NA	NA	136	18.9	17.3
American Samoa	25	10.5^a^	8.4 (4.9–12.0)^a^ 8.0 (4.6–11.4)^b^	NA	NA	NA	NA	NA	NA	NA
CNMI	43	16.0^a^	11.8 (7.4–16.2)^a^ 11.1 (7.2–14.9)^b^	NA	NA	NA	NA	NA	NA	NA
Guam	106	13.1^a^	9.5 (7.6–11.3)^a^ 9.2 (7.4–10.9)^b^	19	22.3	26.2	19.0	NA	NA	NA
FSM	121	28.0^a^	22.6 (18.3–26.9)^a^ 20.5 (16.6–24.5)^b^	NA	NA	NA	NA	NA	NA	NA
Palau	19	18.5^a^	12.8 (6.9–18.8)^a^ 13.6 (7.3–19.8)^b^	NA	NA	NA	NA	NA	NA	NA
RMI	170	81.6^a^	68.9 (57.3–80.5)^a^ 69.4 (58.0–80.7)^b^	NA	NA	NA	NA	NA	NA	NA

Note:

According to Cancer in the U.S. Affiliated Pacific Islands 2007–2022 report,

(*) Age-standardized incidence rate (ASR) (for ages over 20 years) was calculated using the US 2000 standard population.

ASR incidence rates were calculated using different population estimates to account for rapid shifts in the population distribution in some jurisdictions: (a) using 2020 population estimates from the PRCCR database for each jurisdiction as denominator; and (b) using 2022 population estimates from the United Nations Population Division [18].

(α) ASR was calculated using cases estimated by the GLOBOCAN 2022 for Guam, using the US 2000 Standard Population.

(β) ASR was standardized to Segi World Standard Population by GLOBOCAN 2022; The GLOBOCAN used population-weighted averages of estimated incidence rates from the US Pacific Islands (2007–2012) https://gco.iarc.fr/today/en/data-sources-methods-by-country-detailed?tab=6

(∞) ASR was standardized to Segi World Standard Population by Cancer incidence in Five Continents Vol. XI (Bray et al#, 2021);

The age-standardized cervical cancer incidence rate was 10.8 per 100,000 women in the US; NA: Data are not available

Abbreviations: ASR: Age-standardized incidence rates; CI: Confidence interval; CNMI: Commonwealth of the Northern Mariana Islands; FSM: Federated State of Micronesia; GLOBOCAN: Global Cancer Observatory; IARC: International Agency Research for Cancer; PRCCR: Pacific Regional Cervical Cancer Registration; RMI: Republic of the Marshall Islands; USAPI: US-Affiliated Pacific Island;

**Table 4:** Status of HPV vaccination in the US-Affiliated Pacific Islands.

Jurisdiction	Program type^1,2^	Year started^1^	Delivery strategy^3^	Vaccine type	Target age (years)^1^	Dose schedule^1^

**American Samoa**	Jurisdiction-wide	2011	School based and health facility based	HPV4/9	9–17	Started at three doses, then switched to two doses in 2016
**CNMI**	Jurisdiction-wide	2007	School based and health facility based	HPV4/9	9–17	Started at three doses, then switched to two doses in 2016
**Guam**	Jurisdiction-wide	2007	School based and health facility based	HPV4/9	9–18	Started at three doses, then switched to two doses in 2016
**FSM**	Jurisdiction-wide	2009 (Yap, Kosrae, Pohnpei); 2016 (Chuuk)	School based	HPV4/9	9–12 (core) 9–18 (expanded)	Started at three doses, then switched to two doses in 2016
**Palau**	Jurisdiction-wide	2008	School based	HPV4/9	9–17	Started at three doses, then switched to two doses in 2016
**RMI**	Jurisdiction-wide	2009	School based	HPV4/9	9–17	Started at three doses, then switched to two doses in 2016

Data Sources:

1 World Health Organization [56] [15],

2 PATH [57],

3 Bruni, Diaz [54]

Note: Most jurisdictions started with three-dose quadrivalent vaccine (HPV4) and switched to two-dose nonavalent vaccine (HPV9) when made available in 2016 [13];

The jurisdictions were listed by three flag-territories (American Samoa, CNMI, and Guam) and three freely associated states (FSM, Palau, and RMI); Abbreviations; CNMI: Commonwealth of the Northern Mariana Islands; FSM: Federated State of Micronesia; HPV: Human Papillomavirus; RMI: Republic of the Marshall Islands; US: The United States

**Table 5:** Up-to-date cervical cancer screening recommendation and coverage in the US-Affiliated Pacific Islands.

Up-to-date cervical cancer screening recommendation						Screening coverage
		
Jurisdictions	Screening guidelines	Recommended screening test	Screening target ages (years)	Screening intervals	Screening coverage (%) Data source: BRFSS, NCD Hybrid survey, STEPS	Cervical cancer screening coverage either by cytology or HPV testing (%) ~ Data sources: HRSA database [59] for USAPI and NCI [60]

**American Samoa**	USPSTF 2018 [61], ASCCP 2021[62]	Cytology	21–65	3-year	Past 3 years- 29.3%, Never screened: 59.2% *American Samoa NCD Hybrid survey, 2018* [63] *	2018: 14.3% 2019: 47.1% 2020: 2.2% 2021: 15.7% 2022: 2.0%
**CNMI**	USPSTF 2018 [61], ASCCP 2021[62]	Cytology	21–65	3-year	Past 3 years- 43.2% Never screened: 17.9% *CNMI NCD Hybrid survey, 2016* [64] *	2018: Not available 2019: 14.2% 2020: 35.7% 2021: 43.0% 2022: 46.1%
**Guam**	USPSTF 2018 [61], ASCCP 2021 [62]	Cytology Co-testing	21–65 30–65	3-year 5-year	Past 3 years 67.8% Never screened 32.2% *BRFSS, 2018* [65] *	2018: 40.0% 2019: 47.1% 2020: 65.7% 2021: 48.6% 2022: 57.1%
**FSM**	FSM Standard of Care, 2015 [66]	Core resource: 3-year VIA Expanded resource: 5-year VIA screen and treat 3-year cytology	25–45 25–45 21–65	5-year 5-year 3-year	Pohnpei: Past 3 years 28.1% Never screened: 51.8% *Pohnpei NCD Hybrid survey, 2019* [67] # Kosrae: Past 3 years 38.5%, Never screened: 30.7% *Kosrae NCD Hybrid survey, 2019* [29] # Chuuk Past 3 years 13.1%, Never screened: Not available *Chuuk STEPS, 2016* [68] #	2018: 31.0% 2019: 28.9% 2020: 41.4% 2021: 18.1% 2022: 14.6%
**Palau**	USPSTF 2018 [61], ASCCP 2021 [62]	Cytology	21–64	3-year	Past 3 years 60.3%, Never screened: 21.1% *Palau NCD Hybrid survey, 2017* [69] *	2018: 49.0% 2019: 44.9% 2020: 36.7% 2021: 38.5% 2022: 36.9%
**RMI**	National Cancer Control Plan 2017— 2022 [70]	2012: VIA or cytology 2017–2022: VIA		2-year	Past 3 years 33.6%, Never screened: 54.2% *RMI NCD Hybrid survey, 2018* [71] #	2018: 27.1% 2019: 15.7% 2020: 21.4% 2021: 12.9% 2022: 10.0%
**US**	USPSTF 2018 [61], ASCCP 2021 [62]	Cytology Co-testing	21–65 30–65	3-year 5-year	Past 3 years - 80.2%, Never screened: 19.8% *BRFSS, 2018* [72]^	2018: 80.8% 2019: 73.5% 2020: Not available 2021: 72.4% 2022: Not available

Note:

* % of women aged 21–65 years who have or never had a Pap smear in the past three years

# % of women aged 21–65 years who have or never had a Pap or VIA in the past three years

^ 2018 BRFSS Survey Data is the source for this data collected by the BRFSS sponsored by the Centers for Disease Control and Prevention. Data for the US is a median and not a percent. https://www.cdc.gov/brfss/brfssprevalence/

~ According to the definition of cervical cancer screening rates in Health Resources Services Administration (HRSA) https://ecqi.healthit.gov/ecqm/ec/2024/cms0124v12, cervical cancer screening rate in HRSA was calculated as the percentage of women 23–64 years of age who were screened for cervical cancer using either of the following criteria: 1) Women age 21–65 years who had cervical cytology performed within the last 3 years; 2) Women age 30–65 years who had cervical human papillomavirus (HPV) testing performed within the last 5 years.

For the US cervical cancer screening data, up-to-date cervical cancer screening is defined as having a Pap test within the past 3 years for all women aged 21–65 years, with or without an HPV test in the past 5 years for women aged 30–65 years

The jurisdictions were listed by three flag-territories (American Samoa, CNMI, and Guam) and three freely associated states (FSM, Palau, and RMI)

Abbreviations: CI: Confidence interval; CNMI: Commonwealth of the Northern Mariana Islands; FSM: Federated State of Micronesia; HPV: Human Papillomavirus; HRSA: Health Resources Services Administration; NCD: Non-Communicable-Disease; NCI: National Cancer Institute; RMI: Republic of the Marshall Islands; VIA: visual inspection of the cervix after acetic acid application; STEPS: WHO STEPwise approach to NCD risk factor surveillance; US: The United States; USAPI: US-Affiliated Pacific Island;

**Table 6:** Treatment availability of cervical precancer and cancer in the US-Affiliated Pacific Islands.

Jurisdictions	On-island treatment capacity			Off-island treatment
	Human resources	Precancer treatment availability	Cancer treatment availability	

**American Samoa**	• General surgeon • Obstetrician-Gynecologist • General radiologist	• Has capacity to treat precancer	• General surgery • Chemotherapy (maintenance) • Hospital-based and home-based palliative care (in the early stages of development)	• There is almost none or very limited budget for off-island treatment for diagnostic confirmation, advanced staging techniques, or advanced treatment. • Patients can go off-island for treatment usually on their own expense (some patients have Medicare). • Places receive referrals: Honolulu, the US continent, New Zealand
**CNMI**	• General surgeon • Obstetrician-Gynecologist • Oncologist • Interventional radiologist	• Has capacity to treat precancer	• General surgery • Chemotherapy • Hospital-based and home-based palliative care (in the early to midstages of development)	• There is a limited budget for off-island treatment for diagnostic confirmation, advanced staging techniques, or advanced treatment. • Patients can go off-island for treatment usually on their own expense (some patients have Medicare). • Places receive referrals: Honolulu, Guam, the US continent (California)
**Guam**	• General surgeons • Obstetrician-Gynecologists • Oncologists • General and interventional radiologist	• Has capacity to treat precancer • The PACe project currently expands access to precancer assessment (colposcopy) and treatment (LEEP/LLETZ)[75].	• General surgery • Chemotherapy • Radiotherapy (brachytherapy is limited) • Hormonal therapy (limited) • Hospital-based and home-based palliative care (in the early stages of development)	• Off-island referrals to the Philippines commonly occur. • Places receive referrals: the Philippines and other Asian countries
**FSM**	• General surgeon • Obstetrician-Gynecologist	• Has capacity to treat precancer • The PACe project currently introduces thermal ablation for precancer treatment in Yap – a state of FSM[75].	• General surgery • Chemotherapy (maintenance, limited) • Hospital-based and home-based palliative care (in the early stages of development)	• A limited budget for off-island referral • Referrals are considered only for patients at early diagnosis (5-year survival rate is expected to be more than 50%). • Places receive referrals: the Philippines
**Palau**	• General surgeon • Obstetrician-Gynecologist	• Has capacity to treat precancer	• General surgery • Hospital-based and home-based palliative care (in the early stages of development)	• A limited budget for off-island referral • Referrals are considered only for patients at early diagnosis • Places receive referrals: the Philippines
**RMI**	• General surgeon • Obstetrician Gynecologist • General radiologist	• Has capacity to treat precancer	• General surgery • Hospital-based and home-based palliative care (in the early stages of development)	• A limited budget for off-island referral • Referrals are considered only for patients at early diagnosis (5-year survival rate is expected to be more than 50%). • Places receive referrals: the Philippines

Source: Information in the table were extracted from Cancer in the U.S. Affiliated Pacific Islands 2007–2022, except information regarding precancer treatment from the PACe project; The jurisdictions were listed by three flag-territories (American Samoa, CNMI, and Guam) and three freely associated states (FSM, Palau, and RMI) Abbreviations: CNMI: Commonwealth of the Northern Mariana Islands; FSM: the Federated States of Micronesia; RMI: Republic of the Marshall Island; Ob-Gyn: obstetrician and gynaecologist. LEEP: Loop Electrical Excision Procedure; LLETZ: Large Loop Excision of the Transformation Zone. The PACe project: Pacific Against Cervical cancer

## Data Availability

The data that supports the findings of this study are available in the supplementary material of this article.

## Appendix A. Supporting information

Supplementary data associated with this article can be found in the online version at doi:10.1016/j.canep.2026.103144.
