# Supplementary material for: Cervical cancer burden and elimination readiness in the US-affiliated Pacific Islands: A structured narrative review
Source: Cancer Epidemiol. Author manuscript; Available in PMC 2026 Aug 6. (PMC13446121; doi:10.1016/j.canep.2026.103144)
Supplement: Supplementary file 1 [file NIHMS2192740-supplement-Appendix_A__Supplementary_material.pdf]

Cervical cancer burden and elimination readiness in the US-affiliated Pacific Islands: A structured narrative review

Diep TN Nguyen[1] *, Rubana Islam[2] *, Jin Qin[3], Virginia Senkomago[3], Lee Buenconsejo-Lum[4], Youngju Jeong[4], Sameer V. Gopalani[5], Yoon-Jung Kang[1], Michael David[6,7], Martina Reichhardt[8], James Edilyong[6], Ashley Tippins[3], Enriquito Lu[13], Emi Chutaro[11], Isabel Scarinci[12], Theresa Arriola[9], Kate Simms[1], Karen Canfell[1], Neal Palafox[4].

[1] Cancer Elimination Collaboration, School of Public Health, Faculty of Health and Medicine, the University of Sydney, Sydney, Australia.
[2] Australian Institute of Health Innovation, Centre for Health Informatics, Macquarie university.
[3] Division of Cancer Prevention and Control, Centers for Disease Control and Prevention, Atlanta, United States.
[4] John A. Burns School of Medicine, University of Hawaii.
[5] Department of Health Care Management, College of Health Sciences, University of Sharjah, UAE
[6] Daffodil Centre, Sydney, Australia.
[7] School of Medicine & Dentistry, Griffith University, Gold Coast, Australia
[8] Yap State Department of Health Services, Federated States of Micronesia
[9] Guam Department of Public Health and Social Services
[10] Immunization Services Division, Centers for Disease Control and Prevention, Atlanta, United States
[11] Pacific Island Health Officers' Association (PIHOA)
[12] O'Neal Comprehensive Cancer Center, University of Alabama at Birmingham
[13] Independent Consultant of PACe (Pacific Against Cervical Cancer) project

Corresponding author:

Email: Diep.t.nguyen@sydney.edu.au

(*) Joint first authors

Disclaimer

The findings and conclusions in this report are those of the authors and do not necessarily represent the official position of the Centers for Disease Control and Prevention.

Appendix

Abbreviations

ASR: Age-standardized incidence rates;
CI: Confidence interval;
CNMI: Commonwealth of the Northern Mariana Islands;
FSM: Federated State of Micronesia;
HPV: Human Papillomavirus;
RMI: Republic of the Marshall Islands;
STEPS: WHO STEPwise approach to NCD risk factor surveillance;
US: The United States;
USAPI: US-Affiliated Pacific Island;
VIA: visual inspection of the cervix after acetic acid application;

Introduction

Table A1. GDP and Population of US-Affiliated Pacific Islands

USAPI	Area (sq km) (1)	GDP per capita (US$) (2)	Population size 2023 (3)	Average Annual Population growth rate (3)
American Samoa	224	$15,743	44,620	-1.74
CNMI	464	$17,302	51,295	-0.35
FSM	702	$3,855	100,319	-0.70
Guam	544	$35,904	169,330	0.13
RMI	181	$7,227	80,966	1.30
Palau	459	$15,211	21,779	0.39

Figure A1. Map of the US-Affiliated Pacific Islands

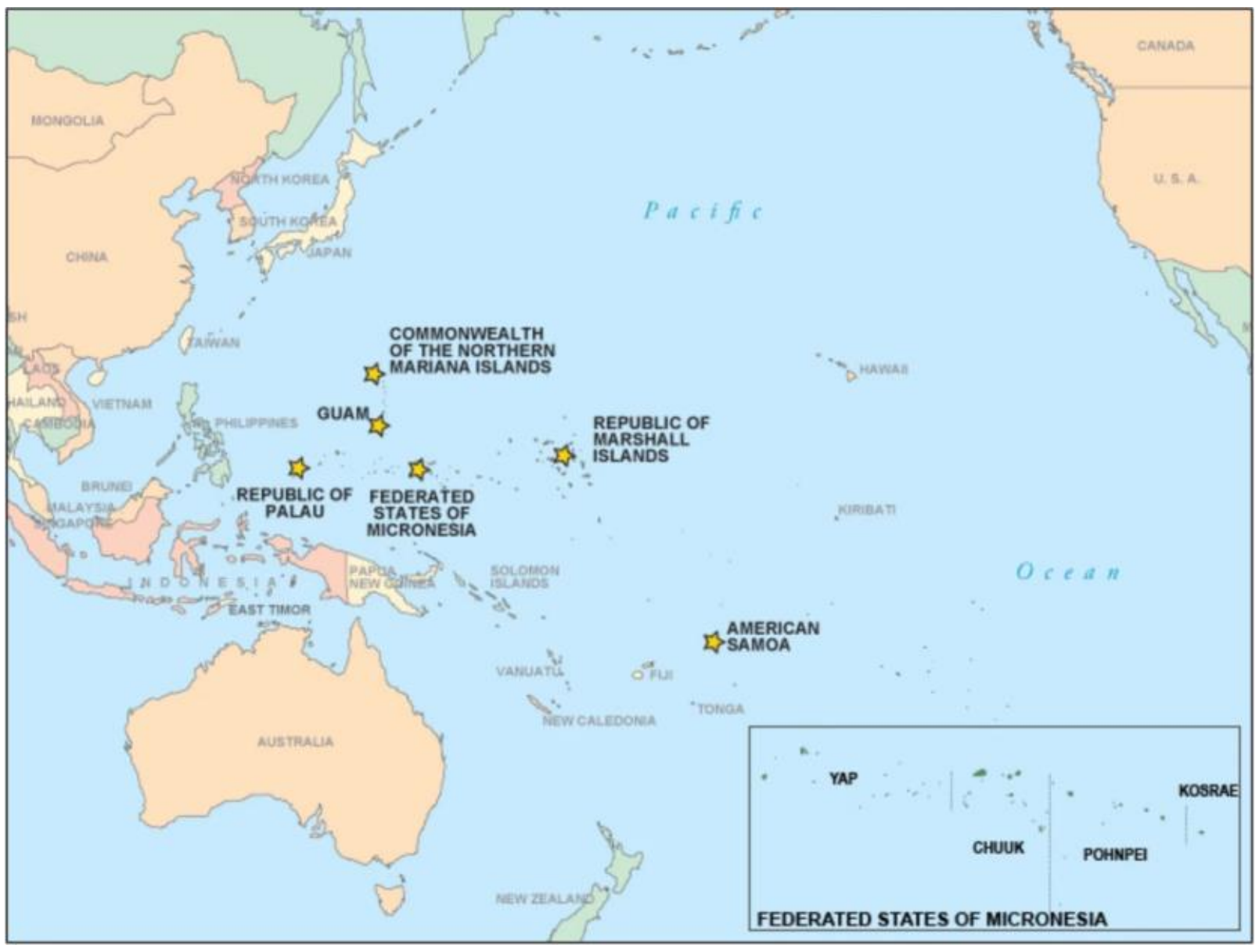

Source: (4)

Methods

Figure A2: Review methods by theme

Commented [DN1]: Added to address comment 5

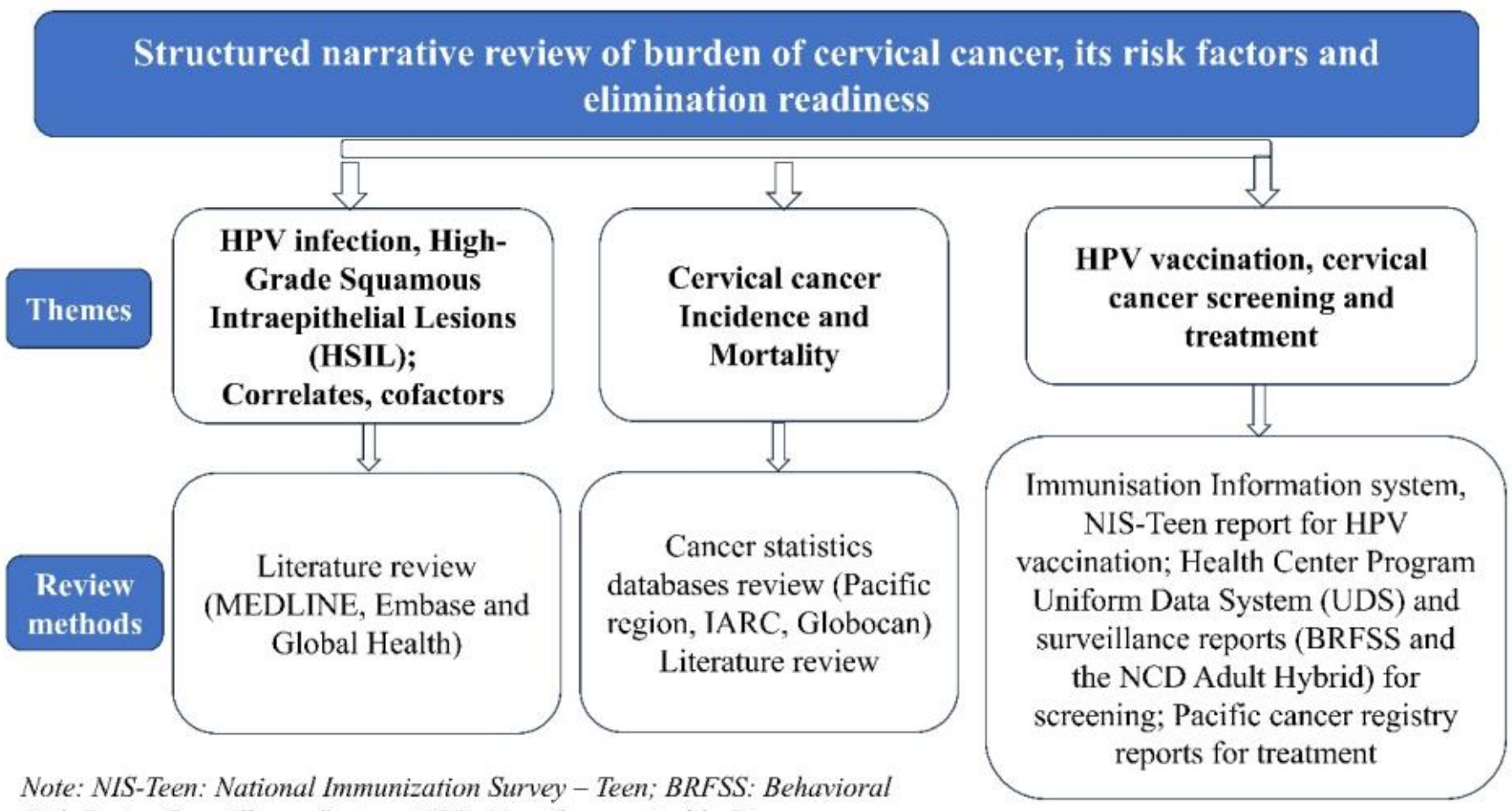

Note: NIS-Teen: National Immunization Survey – Teen; BRFSS: Behavioral Risk Factor Surveillance System; NCD: Non-Communicable Disease

Literature review on the prevalence of HPV infection, precancerous lesions, and correlates of HPV infection and cofactors for invasive cervical cancer progression in the USAPI

Research question

Theme 1: Prevalence of HPV infection and HSIL
PICO question: What is the prevalence of cervical HPV infection among general female population in the USAPI? What is the prevalence of cervical HSIL among general female population in the USAPI?

Population	Outcomes
Female population without symptoms of HPV infection or cervical pre-cancer or cancer in the USAPI	HPV prevalence, LSIL and HSIL prevalence

Table A2. Inclusion and exclusion criteria for research question 1 (Theme: Prevalence of HPV infection and HSIL)

Selection criteria	Inclusion	Exclusion
**Study type**	Prevalence/Rate	
**Study design**	- Population-based cross-sectional studies<br>- Systematic review of above	Comment articles
**Study population**	General female population in the USAPI<br><br>Random samples of general female population in states or jurisdictions in the USAPI.	Study included participants with symptoms or diagnosis with cervical precancer or cervical invasive cancer;
**Outcomes**	Prevalence of cervical HPV infection (any HPV, high-risk HPV);<br><br>Prevalence of cervical high-grade squamous intraepithelial lesions (HSIL)	Studies do not report the prevalence of cervical HPV infection<br>Studies do not report the prevalence of HSIL
**Language**	English	Studies reported in other languages
**Publication period**	Studies published after January 1990 and before July 2025	Studies published before 1990

Theme 2: Correlates and cofactors

PICO question 2.1: In the USAPI general female and male population (where applicable), at which age do people usually commence their first sexual activities? How many lifetime sex partners do they have? How much is the rate of sexual-transmitted infection among general population in the USAPI? Are these factors associated with the transmission of HPV infection in the USAPI?

PICO question 2.2: In the USAPI general female and male population (where applicable), what are the prevalence of oral contraceptive use, smoking, parity, or early age at full term pregnancy? Are these factors associated with the progression from HPV infection to invasive cervical cancer in the USAPI?

**Population**	**Outcomes**
2.1 General female and male population in the USAPI	age at sexual debut, number of lifetime sex partners, STI exposure
2.2 General female and male population in the USAPI	oral contraception, smoking, parity and early age at first full term pregnancy

Table A3. Inclusion and exclusion criteria for research question 2 (Theme: Prevalence of correlates and cofactors)

**Selection criteria**	**Inclusion**	**Exclusion**
**Study type**	Risk factors	
**Study design**	- Cohort or nest case-control studies<br>- Population-based cross-sectional or hospital-based case-control studies<br>- Systematic review of above	Comment articles
**Study population**	- General female and male population in the USAPI	Studies included
**Exposure**	Independently specify the exposure to<br>- Correlates: Age at sexual debut, number of lifetime sex partners, status of STI<br>- Cofactors: oral contraceptive use, smoking, parity, early age at first full term pregnancy	Do not specify the exposure factors
**Comparator/Reference group**	- Do not clearly study the association between each of the correlates and cervical HPV infection.<br>- Do not clearly study the association between each of the cofactor and invasive cervical cancer.	
**Outcomes**	- Independently confirmed the association between each of correlates and cervical HPV infection or reported the prevalence of each factor.	Do not report on the association or prevalence/rate of each exposure factor

	- Independently confirmed the association between each of cofactors and cervical invasive cancer or reported the prevalence of each factor	
**Language**	English	Studies reported in other languages
**Publication period**	Studies published after January 1990 and before July 2025	Studies published before 1990

Search Strategy:

1. (USAPI or "American Samoa" or "FSM" or "Federated States of Micronesia" or Palau, Guam, CNMI or "Commonwealth of the Northern Mariana Islands" or "Marshall Islands" or Guam).mp. [mp=title, book title, abstract, original title, name of substance word, subject heading word, floating sub-heading word, keyword heading word, organism supplementary concept word, protocol supplementary concept word, rare disease supplementary concept word, unique identifier, synonyms]
2. (HPV or "Human papilloma virus" or "Papillomavirus Infections" OR "HPV infection" OR "cervical cancer" or "HPV screen*" or “PAP smear” or "Squamous Intraepithelial Lesions of the Cervix" OR "cervical cancer screen*" or "HPV vaccin*" or "HSIL" or "Cervical Intraepithelial Neoplasia" OR "Uterine Cervical Neoplasms" OR "Uterine Cervical Dysplasia" OR "Squamous Intraepithelial Lesions of the Cervix" or "CIN" OR "cervical cancer" OR "cervical precancer" OR "uterine cervical neoplasm*") [mp=title, book title, abstract, original title, name of substance word, subject heading word, floating sub-heading word, keyword heading word, organism supplementary concept word, protocol supplementary concept word, rare disease supplementary concept word, unique identifier, synonyms]
3. ("sex* behav*" or "sex* partner*" or "multiple partner*" or STI or "sexually transmitted diseases" or "age at first sex* intercourse" or "age at first intercourse" or "age of sexual debut" or "sexual initiation").mp. [mp=title, book title, abstract, original title, name of substance word, subject heading word, floating sub-heading word, keyword heading word, organism supplementary concept word, protocol supplementary concept word, rare disease supplementary concept word, unique identifier, synonyms]
4. (Smok* or "oral contraceptive" or "parity" or "multiparity" or "number of children" or "number of pregnancy" or "first pregnancy" or "oral contraceptive" or Contraceptiv* or "Oral Contraceptives" or "Oral pill" or "Hormonal Contraceptives*" or "Oral, Combined/ or contraceptive pill" or "Contraception/ family planning" or "Family Planning Services" or "age adj4 pregnanc*").mp. [mp=title, book title, abstract, original title, name of substance word, subject heading word, floating sub-heading word, keyword heading word, organism supplementary concept word, protocol supplementary concept word, rare disease supplementary concept word, unique identifier, synonyms]

   Final search: 1 AND (2 OR 3 OR 4)

Note: The review questions were developed following the Condition, Context, Population (CoCoPop) framework for reviews on prevalence or incidence studies (5).

Correlates and cofactors

Figure A3: Sexual behaviour pattern among adolescents aged 13-19 years in the US-Affiliated Pacific Islands

a. Ever had sex

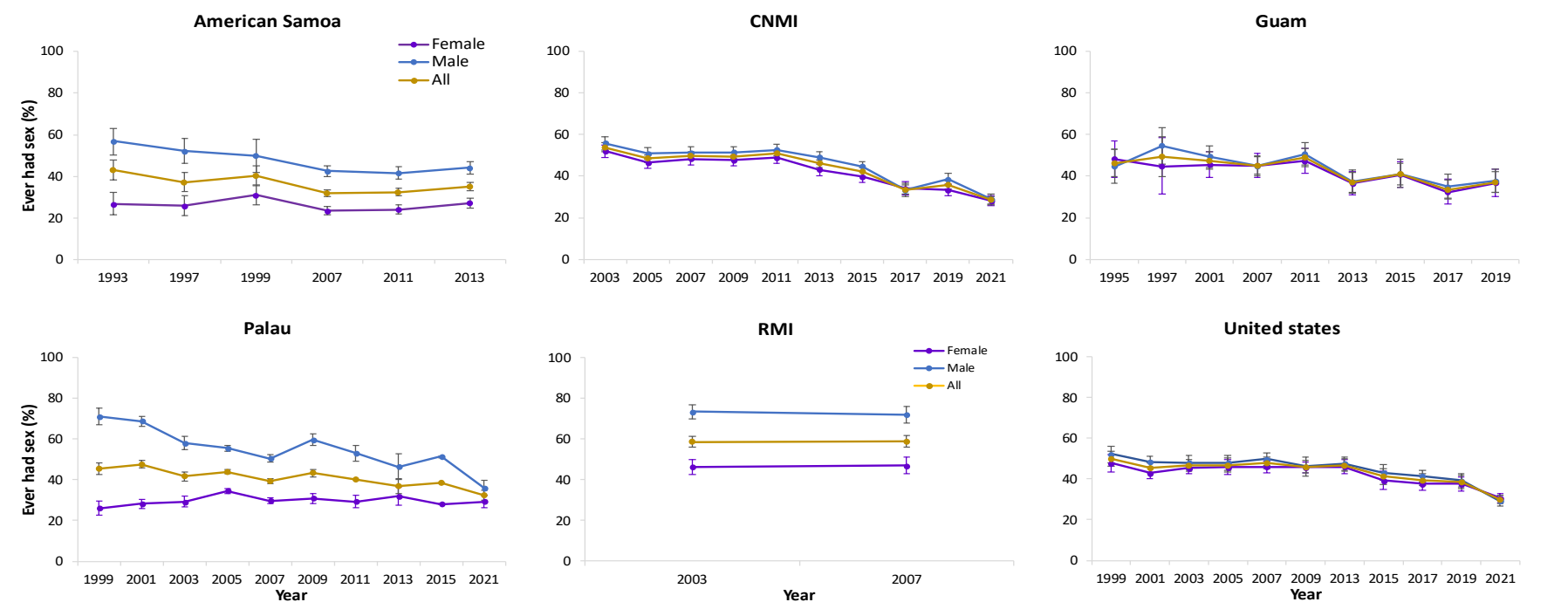

b. Sexual debut by age 13 years

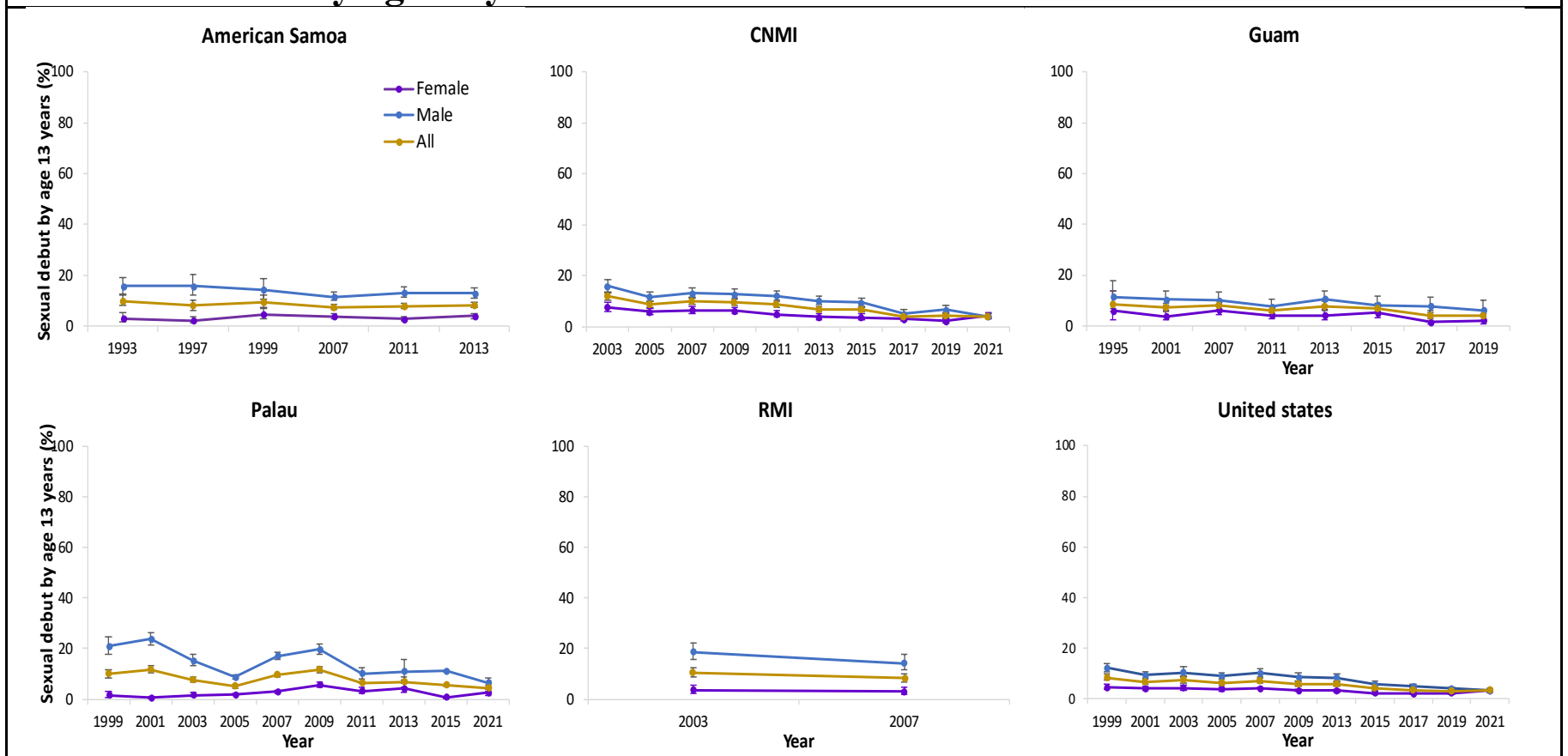

c. Had more than four sexual partners

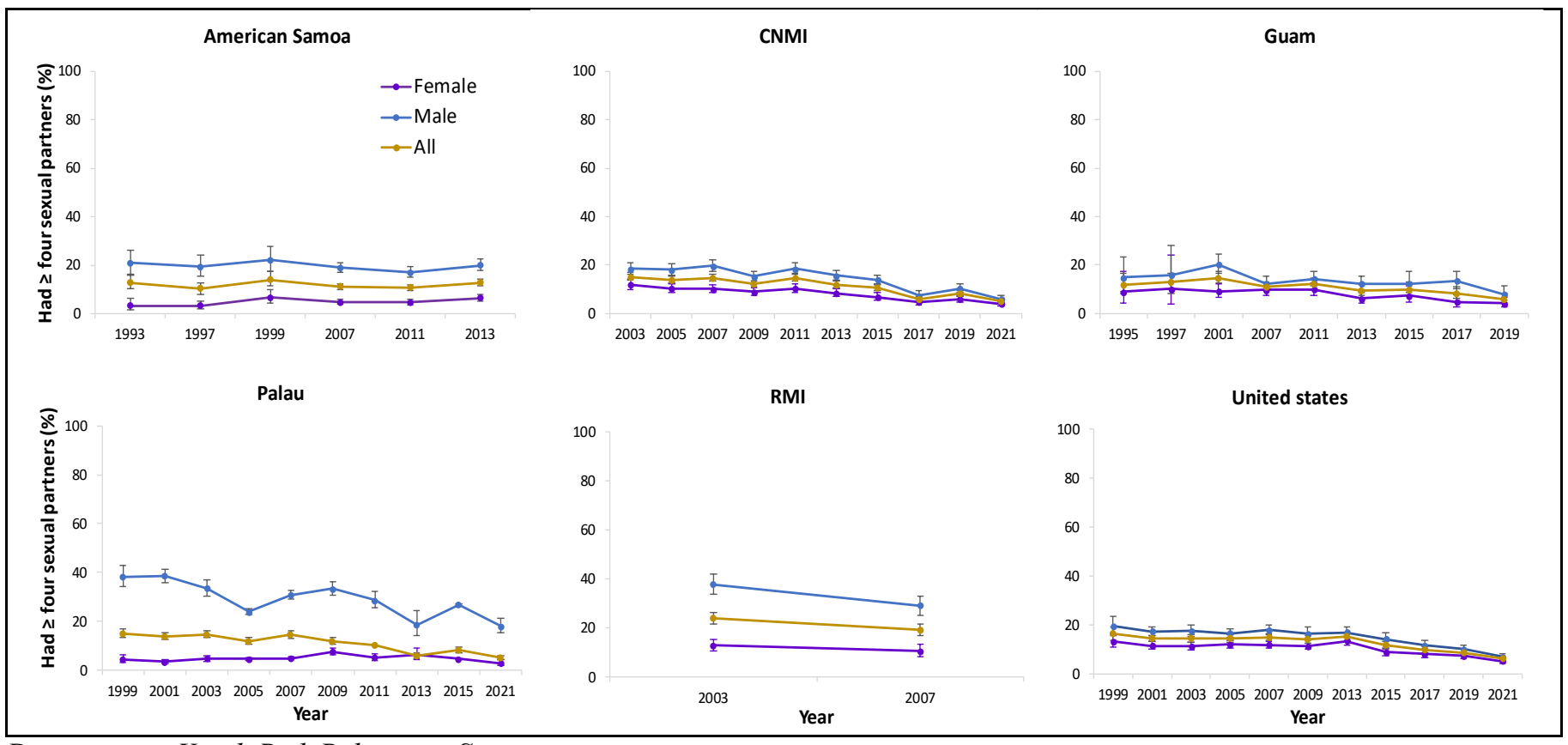

Data source: Youth Risk Behaviour Surveys

Figure A4: Smoking prevalence among adolescents aged 13-19 years in the US-Affiliated Pacific Islands

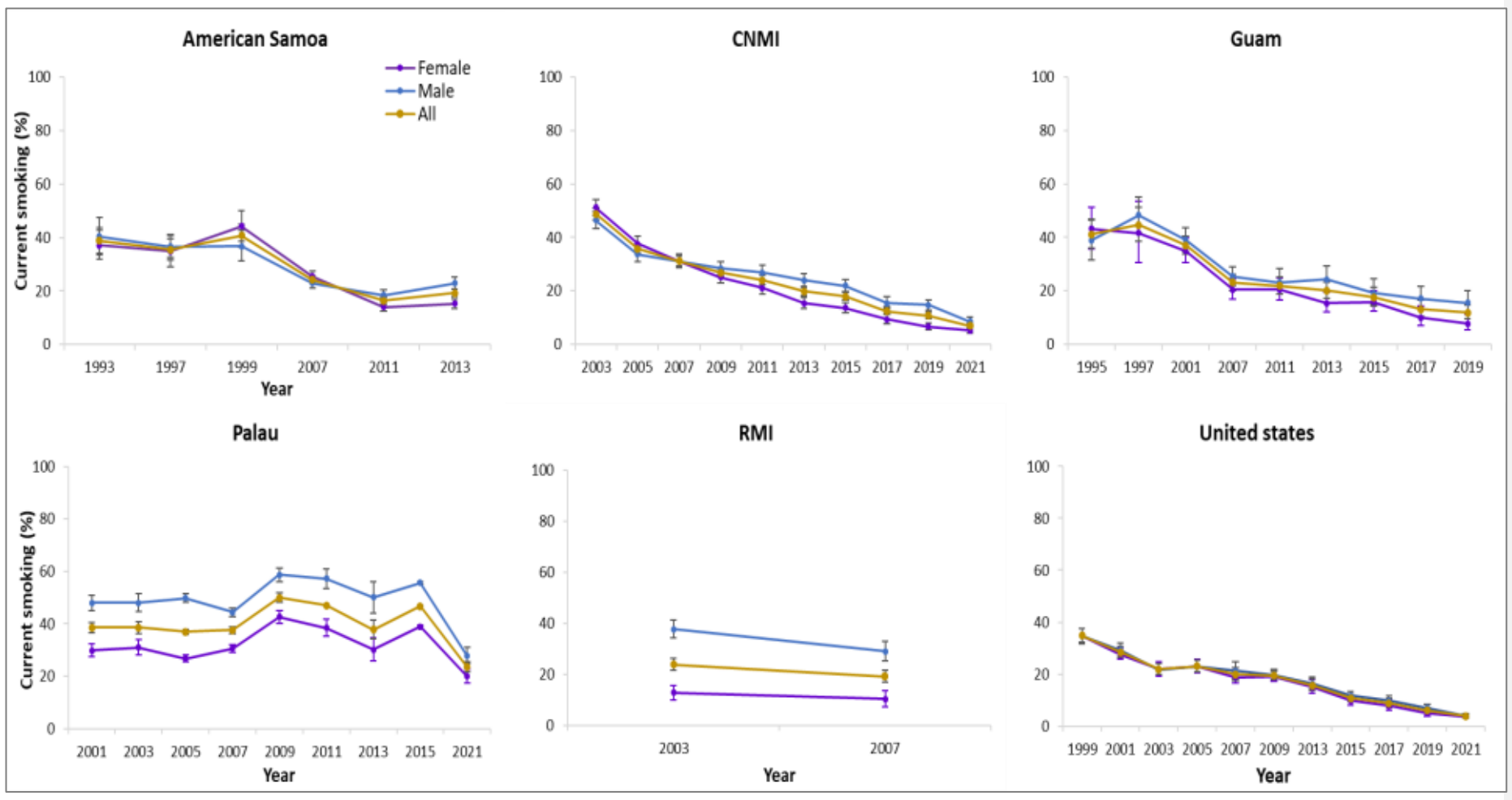

Data source: Youth Risk Behaviour Surveys
Note: Current smoking estimated as currently smoked cigarettes (on at least 1 day during the 30 days before the survey)

Table A4: FSM sexual behaviour and smoking prevalence (%), from Rapid Youth Surveys 2016-2023

FSM states	Ever had sexual intercourse % (95% CI)	Sexual debut by age 13 years, %	Having more than 4 sexual partners, %	Current smoking % (95% CI)
**Yap**				
Yap Youth Survey, 2016 (6)	46.2 (42.4-50.0)	23.5	30.4	39.0 (35.5-42.7)
Yap Youth Survey, 2021 (7)	38.9(34.8-43.1)	19.2	33.8	36.7 (32.7-40.9)
Yap Youth Survey, 2023-2024(8)	34.7 (30.7-39.0)	15.9	30.4	40.4 (36.2-44.7)
**Chuuk**				
Chuuk Rapid Youth Survey 2017 (9)	..	..	..	19.4 (16.9-21.9)
Chuuk Rapid Youth Survey 2019 (10)	22.1 (19.8-24.4)	33.9	23.0	17.4 (15.3-19.4)
Chuuk Rapid Youth Survey 2021(11)	37.5(34.6-40.4)	23.2	21.6	23.3 (20.9-26.0)
**Kosrae**				
Kosrae Rapid Youth Survey 2017(12)	..	..	..	24.6 (21.0-28.20)
Kosrae Rapid Youth Survey2019(13)	..	..	..	25.9 (21.9-29.7)
Kosrae Rapid Youth Survey2021(14)	..	..	..	23.5 (19.5-27.9)
Kosrae Rapid Youth Survey2023	..	..	..	28.6 (24.3-33.3)
**Pohnpei**				
Pohnpei Rapid Youth Survey 2017(15)	42.8 (40.6-45.0)	32.7	26.5	30.8 (28.8-32.9)
Pohnpei Rapid Youth Survey 2019(16)	38 (35.7-40.3)	..	20.4	30.6 (28.4-32.8)
Pohnpei Rapid Youth Survey 2023/2024(17)	35.4 (33.3-37.6)	32.5	25.0	35.2(33.1-37.4)
**FSM**				
FSM youth NCD risk factors 2017(18)	..	..	..	28.9 (27.5-30.3)

Note: ".." indicates that no data are available.
Source: Rapid Youth Surveys in four states of FSM

Table A5: Cigarette smoking among adolescents aged 13-15 years in Guam, Palau and FSM from Global Youth Tobacco Survey 2012-2020

**USAPI**	**Crude prevalence**		**Adjusted prevalence**		**Adjusted prevalence difference, (95% CI)**
	Base wave, % (95% CI)	Latest wave, % (95% CI)	Base wave, % (95% CI)	Latest wave, % (95% CI)	
FSM	27.3 (27.3 - 27.3)	20.6 (18.7 - 22.6)	25.2 (23.7 - 26.8)	21.8 (19.4 - 24.4)	−3.5 (−5.8 - −1.2)
Guam	14.9 (11.7 - 19.0)	10.6 (8.5 - 13.1)	12.1 (10.3 - 14.3)	12.1 (10.3 - 14.3)	0 (NA)
Palau	32.1 (32.1 - 32.1)	32.6 (28.2 - 37.2)	29.9 (27.4 - 32.6)	35.0 (30.4 - 39.8)	5.0 (0.7 - 9.3)

Source: Data were extracted from Njie et.al., 2023, Table 1: Changes in the Prevalence of Cigarette Smoking among Youth aged 13-15 years, by Country, Global Youth Tobacco Survey 2012-2020(19)

List of included studies

Table A6 List of included studies

Study		Author, year	Sample population	Age (years)	Sample size	Had sex before 13 years of age (% and 95% CI if available)	Had 4 or more sexual partners (% and 95% CI if available)	Infection with STIs
**American Samoa**								
Youth Risk Behaviour Surveillance System (YRBSS)	1993	CDC, USA(20)	Grade 9-12 attending female students	14-18	425	3.1 (1.8–5.3)	3.3 (1.7–6.3)	NA
	1997				545	2.3 (1.4–3.7)	3.2 (2.0–5.0)	
	1999				415	4.6 (2.9–7.3)	6.6 (4.3–10.0)	
	2007				1463	3.8 (3.0–4.8)	4.7 (3.8–5.8)	
	2011				1241	2.8 (2.1–3.8)	4.7 (3.7–6.0)	
	2013				1249	4.0 (3.1–5.1)	6.5 (5.3–8.0)	
Cervical and Anal Human Papillomavirus Infection in Adult Women in American Samoa, 2008-2009		Hernandez, 2013 (21)	Not currently pregnant and at least 6 months postpartum, and having an intact cervix (no prior hysterectomy).	18-82	211	Age at sexual debut:<br><17 12%<br>19-19 30%<br>20-24 41%<br>≥25 17%	NA	NA
Sexually Transmitted Disease Surveillance 2021 (Chlamydia)	2018	CDC, USA(22)	NA	0-65+	NA	NA	NA	208.5/100,000
	2019							333.8/100,000
	2020							349.3/100,000
	2021							176.2/100,000
	2018		NA	NA	NA	NA	NA	15.7/100,000

Study		Author, year	Sample population	Age (years)	Sample size	Had sex before 13 years of age (% and 95% CI if available)	Had 4 or more sexual partners (% and 95% CI if available)	Infection with STIs
Sexually Transmitted Disease Surveillance 2021 (Gonorrhoea)	2019	CDC, USA(23)						8.2/100,000
	2020							46.3/100,000
	2021							21.5/100,000
**CNMI**								
Youth Risk Behaviour Surveillance System (YRBSS)	2003	CDC USA(20)	Grade 9-12 attending female students	14-18	934	7.6 (6.1–9.4)	11.8 (9.8–14.0)	NA
	2005				1,047	5.8 (4.7–7.2)	10.3 (8.7–12.1)	
	2007				1,035	6.5 (5.3–8.0)	10.1 (8.5–11.9)	
	2009				1,097	6.2 (5.0–7.6)	8.9 (7.5–10.6)	
	2011				1,120	4.9 (3.8–6.3)	10.3 (8.7–12.1)	
	2013				1,036	3.8 (2.9–5.1)	8.4 (7.0–10.2)	
	2015				1,075	3.7 (2.8–5.0)	6.8 (5.5–8.5)	
	2017				839	3.1 (2.1–4.4)	4.7 (3.4–6.3)	
	2019				1,012	2.2 (1.5–3.1)	5.9 (4.7–7.4)	
	2021				995	4.2 (3.2–5.5)	4.0 (3.0–5.4)	
Sexually Transmitted Disease	2017	CDC, USA(22)	Women	0-65+	NA	NA	NA	310.7/100,000
	2018							776.0/100,000
	2019							823.7/100,000

Study		Author, year	Sample population	Age (years)	Sample size	Had sex before 13 years of age (% and 95% CI if available)	Had 4 or more sexual partners (% and 95% CI if available)	Infection with STIs
Surveillance 2021 (Chlamydia)	2020							591.8/100,000
	2021							696.5/100,000
Sexually Transmitted Disease Surveillance 2021 (Gonorrhoea)	2017	CDC, USA(23)	Women	0-65+	NA	NA	NA	45.0/100,000
	2018							65.7/100,000
	2019							53.3/100,000
	2020							8.2/100,000
	2021							45.3/100,000
**FSM**								
Rapid Youth Survey (RYS) 2016-2021 (in Yap/Chuuk/ Pohnpei)		Chuk DHS (24); Pohnpei DHS (24) Yap DHS (7)	Grade 9-12 attending female students	12-19	295-995	9%-23%	34%-70%	NA
Randomized controlled trial evaluating the utility of urine HPV DNA for cervical cancer screening in a Pacific Island population		Hernandez, 2018 (25)	Yap women	21-65	217	NA	67.5% (>2 partners)	17.5% (any STIs)
Kosrae Adult hybrid survey 2017		Kosrae DHS (26)		≥18	297	NA	NA	Gonorrhoea 1.4% Chlamydia 2.4%
**Guam**								

Study		Author, year	Sample population	Age (years)	Sample size	Had sex before 13 years of age (% and 95% CI if available)	Had 4 or more sexual partners (% and 95% CI if available)	Infection with STIs
Youth Risk Behaviour Surveillance System (YRBSS)	1995	CDC, USA(20)	Grade 9-12 attending female students	14-18	134	5.9 (2.4–13.7)	8.9 (4.3–17.4)	NA
	1997				163	NA	10.1 (3.8–24.2)	
	2001				673	3.8 (2.3–6.0)	9.1 (6.8–12.2)	
	2007				744	6.3 (4.6–8.7)	9.7 (7.4–12.5)	
	2011				682	4.2 (2.8–6.4)	9.8 (7.6–12.5)	
	2013				608	4.1 (2.6–6.6)	6.2 (4.4–8.5)	
	2015				500	5.1 (3.2–8.1)	7.3 (4.7–11.3)	
	2017				485	1.5 (0.7–3.5)	4.7 (2.7–8.0)	
	2019				487	2.1 (0.9–4.7)	4.2 (2.7–6.6)	
Sexually Transmitted Disease Surveillance 2021 (Chlamydia)	2017	CDC, USA (22)	Women	0-65+	NA	NA	NA	926.6/100,000
	2018							822.7/100,000
	2019							1,070.9/100,000
	2020							773.0/100,000
	2021							639.9/100,000
Sexually Transmitted Disease Surveillance	2017	CDC, USA (23)	Women	0-65+	NA	NA	NA	175.6/100,000
	2018							181.3/100,000
	2019							132.1/100,000
	2020							119.7/100,000

Study		Author, year	Sample population	Age (years)	Sample size	Had sex before 13 years of age (% and 95% CI if available)	Had 4 or more sexual partners (% and 95% CI if available)	Infection with STIs
2021 (Gonorhhoea)								
National Center for HIV, Viral Hepatitis, STD, and TB Prevention surveillance data (HIV diagnoses in men and women aged 13 and older)	2008	CDC, USA (27)	Men and women	13+	NA	NA	NA	3.0/100,000
	2009							2.2/100,000
	2010							4.9/100,000
	2011							0.8/100,000
	2012							5.7/100,000
	2013							0.8/100,000
	2014							2.4/100,000
	2015							4.8/100,000
	2016							3.2/100,000
	2017							3.2/100,000
	2018							5.5/100,000
	2019							8.6/100,000
**RMI**								
Youth Risk Behaviour Surveillance System (YRBSS)	2003	CDC USA (20)	Grade 9-12 attending female students	14-18	268	3.7 (2.5–5.5)	12.8 (10.6–15.4)	NA
	2007				435	3.0 (1.8–4.9)	10.4 (8.1–13.1)	
**Palau**								
Youth Risk Behaviour Surveillance System (YRBSS)	1999	CDC, USA (20)	Grade 9-12 attending female students	14-18	237	1.7 (0.9–3.0)	4.5 (3.2–6.4)	NA
	2001				219	0.6 (0.4–1.1)	3.5 (2.6–4.5)	
	2003				255	1.7 (1.0–2.6)	4.8 (3.7–6.1)	
	2005				277	1.8 (1.5–2.2)	4.7 (4.1–5.3)	
	2007				331	3.0 (2.5–3.6)	4.8 (4.2–5.6)	

Study		Author, year	Sample population	Age (years)	Sample size	Had sex before 13 years of age (% and 95% CI if available)	Had 4 or more sexual partners (% and 95% CI if available)	Infection with STIs
	2009				265	5.6 (4.6–6.8)	7.7 (6.5–9.2)	
	2011				213	3.3 (2.3–4.7)	5.2 (3.9–6.9)	
	2013				144	4.2 (2.7–6.4)	6.3 (4.4–9.0)	
	2015				241	0.8 (0.8–0.9)	4.6 (4.4–4.8)	
	2021				178	2.6 (1.6–4.0)	2.9 (1.9–4.3)	
**ALL USAPIs**								
Diagnoses of HIV Infection in the United States and Dependent Areas	2015	CDC, USA (27)	NA	13-65+		NA	NA	5.4/100,000
	2016					NA	NA	6.1/100,000
	2017					NA	NA	5.6/100,000
	2018					NA	NA	5.0/100,000
	2019					NA	NA	4.9/100,000
	2020					NA	NA	3.9/100,000

Cervical cancer cases, incidence rate, and cancer stages

Table A7 Cervical cancer incidence rates in the USAPI (2007-2022) by different standard populations

USAPI	Cancer cases*	Crude incidence rate/100,000 (95% CI)	ASR incidence /100,000 (95% CI)[(a)]	ASR incidence /100,000 (95% CI)[(b)]	ASR incidence /100,000 (95% CI)[(c)]
**A7.1. Population estimates from the US Census Bureau's International Database were used to estimate crude rates**					
American Samoa	25	10.3 (6.6-15.1)	10.4 (5 - 20.1)	10.7 (5.1 - 20.6)	11.7 (5.6 - 22.3)
CNMI	43	15.8 (11.5 - 21.3)	15.8 (8.5 - 26.8)	16.0 (8.6 - 27.4)	16.8 (9.1 - 28.7)
Guam	106	12.9 (10.5 - 15.6)	12.6 (8.9 - 17.3)	12.5 (8.8 - 17.3)	12.9 (9.1 - 17.9)
FSM	121	24.4 (20.2 - 29.1)	26.2 (18.8 - 35.7)	26.4 (18.8 - 36.2)	27.8 (19.8 – 38.2)
Palau	19	16.9 (10.2 - 26.4)	NA	NA	NA
RMI	170	55.3 (47.3 - 64.3)	60.6 (46.3 - 78.3)	60.5 (46.1 - 78.5)	63.3 (48.1 - 82.3)
**Total**	**484**	**21.5 (19.6 - 23.5)**	**21.3 (18.2 - 24.8)**	**21.3 (18.2 - 24.9)**	**22.2 (18.9 - 25.9)**
**A7.2. Population estimates from the United Nations Population Division were used to estimate crude rates**					
American Samoa	25	10.3 (6.7 - 15.2)	10.4 (5 - 20.2)	10.7 (5.1 - 20.7)	11.7 (5.6 - 22.4)
CNMI	43	16.0 (11.6 - 21.6)	16.3 (8.8 - 27.8)	16.6 (9 - 28.4)	17.6 (9.5 – 30.0)
Guam	106	12.5 (10.3 - 15.2)	12.2 (8.6 - 16.8)	12.2 (8.6 - 16.8)	12.5 (8.9 - 17.3)
FSM	121	25.0 (20.7 - 29.9)	26.3 (18.9 - 35.7)	26.4 (19 - 35.9)	27.7 (19.8 - 37.7)
Palau	19	19.7 (11.9 - 30.8)	NA	NA	NA
RMI	170	85.8 (73.4 - 99.7)	93.3 (70.5 - 122.1)	93.6 (70.4 - 123)	98.2 (73.6 - 129.5)
**Total**	**484**	**22.7 (20.7 - 24.8)**	**22.3 (19.1 – 26.0)**	**22.3 (19.1 - 26.1)**	**23.2 (19.8 – 27.0)**
**A7.3: Population estimates from the Pacific Data Hub were used to estimate crude rates**					
American Samoa	25	10.3 (6.7 - 15.2)	10.4 (5 - 20.1)	10.7 (5.1 - 20.6)	11.7 (5.6 - 22.3)
CNMI	43	16 (11.6 - 21.6)	16.3 (8.8 - 27.8)	16.6 (9 - 28.4)	17.6 (9.5 – 30.0)
Guam	106	12.5 (10.3 - 15.2)	12.2 (8.6 - 16.8)	12.2 (8.6 - 16.8)	12.5 (8.9 - 17.3)
FSM	121	25.0 (20.7 - 29.8)	26.3 (18.9 - 35.6)	26.4 (18.9 - 35.9)	27.6 (19.8 - 37.7)

USAPI	Cancer cases*	Crude incidence rate/100,000 (95% CI)	ASR incidence /100,000 (95% CI)[(a)]	ASR incidence /100,000 (95% CI)[(b)]	ASR incidence /100,000 (95% CI)[(c)]
Palau	19	20.5 (12.3 – 32.0)	NA	NA	NA
RMI	170	85.7 (73.3 - 99.6)	93.1 (70.4 - 121.8)	93.4 (70.3 - 122.7)	98 (73.5 - 129.1)
**Total**	**484**	**22.7 (20.7 - 24.8)**	**22.4 (19.1 - 26.0)**	**22.4 (19.1 - 26.1)**	**23.2 (19.8 - 27.1)**

*Note: *Data source: PRCCR data 2007-2022 (28); ASR: Age-standardized rate*
(a) Age-standardized incidence rate was standardized to Segi World Standard Population, calculated based on PRCCR data 2007-2022;
(b) Age-standardized incidence rate was standardized to 2015 World Standard population, calculated based on PRCCR data 2007-2022;
(c) Age-standardized incidence rate was standardized to US 2000 Standard Population; calculated based on PRCCR data 2007-2022;
ASR was not calculated for Palau following statistical guidelines, given number of observations were less than six in one of age groups. ASR and 95% CI calculation methods were described in Gopalani et al, 2024.(29)

Table A8: Stage distribution at diagnosis of cervical cancer cases

Stage	American Samoa	CNMI	Guam	FSM	Palau	RMI	USAPI
**Stage I**	5 (20.0%)	3 (7%)	19 (17.9%)	31 (18.2%)	10 (8.3%)	1 (5.3%)	69 (14.3%)
**Stage II**	0 (0.0%)	5 (11.6%)	7 (6.6%)	24 (14.1%)	17 (14.0%)	0 (0.0%)	53 (11.0%)
**Stage III &IV**	20 (80.0%)	35 (81.4%)	80 (75.5%)	115 (67.6%)	94 (77.7%)	18 (94.7%)	362 (74.8%)
**Total**	**25 (100%)**	**43 (100%)**	**106 (100%)**	**170 (100%)**	**121 (100%)**	**19 (100%)**	**484 (100%)**

Note: Cancer cases by stages were reported based on Pacific Cancer Registries' data over 2007-2022. The cervical cancer stages were classified following the International Federation of Gynecology and Obstetrics (FIGO) staging system.(30)

References

1. CIA. World factbook: CIA; Nd [Available from: https://www.cia.gov/the-world-factbook/.
2. The World Bank. GDP per capita (current US$): World Bank,; Nd [cited 2023 17 June]. Available from: https://data.worldbank.org/indicator/NY.GDP.PCAP.CD.
3. U.S. Census Bureau. Population estimates and projections for 227 countries and areas 2023 [cited 2023 June 13]. Available from: https://www.census.gov/data-tools/demo/idb/#/country?COUNTRY_YEAR=2023&COUNTRY_YR_ANIM=2023.
4. Hosey GM, Samo M, Gregg EW, Padden D, Bibb SG. Socioeconomic and demographic predictors of selected cardiovascular risk factors among adults living in Pohnpei, Federated States of Micronesia. BMC Public Health. 2014;14(1):1-14.
5. Munn Z, Moola S, Lisy K, Riitano D, Tufanaru C. Methodological guidance for systematic reviews of observational epidemiological studies reporting prevalence and cumulative incidence data. JBI Evidence Implementation. 2015;13(3):147-53.
6. Department of Health Services. Yap Rapid Youth Survey 2016 Report. Yap: DHS, State of Yap; 2016.
7. Department of Health Services. Yap Rapid Youth Survey 2021-2022 Report. Yap: DHS, State of Yap; 2021.
8. Department of Health Services. Yap Rapid Youth Survey 2023-2024 Report. Yap: DHS, State of Yap; 2024.
9. Department of Health Services. Chuuk Rapid Youth Survey 2017 Report. Chuuk: DHS, State of Chuuk; 2017.
10. Chuuk Department of Health Services. Chuuk Rapid Youth Survey 2019 Report. Chuuk: DHS, State of Chuuk; 2019.
11. Chuuk Department of Health Services. Chuuk Rapid Youth Survey 2021 Report. Chuuk: DHS, State of Chuuk; 2021.
12. Department of Health Services. Kosrae Rapid Youth Survey 2017 Report. Kosrae: DHS, State of Kosrae; 2017.
13. Department of Health Services. Kosrae Rapid Youth Survey 2019 Report. Kosrae: DHS, State of Kosrae; 2019.
14. Department of Health Services. Kosrae Rapid Youth Survey 2021 Report. Kosrae: DHS, State of Kosrae; 2021.
15. Department of Health Services. Pohnpei Rapid Youth Survey 2017 Report. Pohnpei: DHS, State of Pohnpei; 2017.
16. Department of Health Services. Pohnpei Rapid Youth Survey 2019 Report. Pohnpei: Department of Health and Social Services, State of Pohnpei; 2019.
17. Department of Health Services. Pohnpei Rapid Youth Survey 2023/2024 Report. Pohnpei: DHS, State of Pohnpei; 2019.
18. Federates States of Micronesia Department of Health Services. FSM Youth NCD Risk Factors 2017 Report. Federates States of Micronesia: DHS, State of Federated States of Micronesia; 2017.
19. Njie GJ, Kirksey Jones C, Jacques N, Adetokun A, Ross J, Owens A, et al. Changes in Tobacco Product Use Among Students Aged 13 to 15 Years in 34 Countries, Global Youth Tobacco Survey, 2012-2020. Preventing chronic disease. 2023;20:E68.
20. Centers for Disease Control and Prevention. Youth Risk Behvaiour Surveillance System. Nd.
21. Hernandez BY, Ka'opua LS, Scanlan L, Ching JA, Kamemoto LE, Thompson PJ, et al. Cervical and anal human papillomavirus infection in adult women in American Samoa. Asia Pacific journal of public health. 2013;25(1):19-31.

22. Centers for Disease Control and Prevention. Chlamydia Among Women — Reported Cases and Rates of Reported Cases by State/Territory and Region in Alphabetical Order, United States, 2017–2021 2021 [Available from: https://www.cdc.gov/std/statistics/2021/tables/3b.htm.
23. Centers for Disease Control and Prevention. Gonorrhea Among Women — Reported Cases and Rates of Reported Cases by State/Territory and Region in Alphabetical Order, United States, 2017–2021 2021 [Available from: https://www.cdc.gov/std/statistics/2021/tables/8b.htm.
24. Department of Health Services. Chuuk Rapid Youth Survey 2021 Report. Chuuk: DHS, State of Chuuk; 2021.
25. Hernandez BY, Tareg AC, Reichhardt M, Agapito A, Zhu X, Sy A, et al. Randomized controlled trial evaluating the utility of urine HPV DNA for cervical cancer screening in a Pacific Island population. Journal of global health reports. 2018;2.
26. Department of Health Services. Adult Hybrid Survey. DHS, Kosrae Federated States of Micronesia; 2019.
27. Centers for Disease Control and Prevention. Diagnoses of HIV Infection in the United States and Dependent Areas 2019 2019 [Available from: https://www.cdc.gov/hiv/library/reports/hiv-surveillance/vol-32/content/tables.html.
28. Pacific Regional Central Cancer Registry (PRCCR). Cancer in the U.S. Affiliated Pacific Islands 2007-2020. 2023.
29. Gopalani SV, Qin J, Baksa J, Thompson TD, Saraiya M, Senkomago V, et al. Cervical Cancer Incidence in the US-Affiliated Pacific Islands. JAMA Oncology. 2024.
30. National Cancer Institute. Cervical Cancer Stages [Internet]. National Cancer Institute; [cited 2025 July 1]. Available from: https://www.cancer.gov/types/cervical/stages.
